# Metagenomic reconstructions of gut microbial metabolism in weanling pigs

**DOI:** 10.1186/s40168-019-0662-1

**Published:** 2019-03-26

**Authors:** Weilan Wang, Huifeng Hu, Ruurd T. Zijlstra, Jinshui Zheng, Michael G. Gänzle

**Affiliations:** 1 Department of Agricultural, Food and Nutritional Science, University of Alberta, 4-10 Ag/For Centre, Edmonton, Alberta T6G 2P5 Canada; 2 State Key Lab of Agricultural Microbiology, Hubei Key Laboratory of Agricultural Bioinformatics, Huazhong Agricultural University, Wuhan, 430070 People’s Republic of China; 3 Hubei University of Technology, College of Bioengineering and Food Science, Wuhan, People’s Republic of China

**Keywords:** Metagenomic, Reconstructions, Weanling pigs, Microbial degradation, Lactose, Fructan, Starch

## Abstract

**Background:**

The piglets’ transition from milk to solid feed induces a succession of bacterial communities, enhancing the hosts’ ability to harvest energy from dietary carbohydrates. To reconstruct microbial carbohydrate metabolism in weanling pigs, this study combined 16S rRNA gene sequencing (*n* = 191) and shotgun metagenomics (*n* = 72).

**Results:**

Time and wheat content in feed explained most of the variation of the microbiota as assessed by 16S rRNA gene sequencing in weanling pigs. De novo metagenomic binning reconstructed 360 high-quality genomes that represented 11 prokaryotic and 1 archaeal phylum. Analysis of carbohydrate metabolism in these genomes revealed that starch fermentation is carried out by a consortium of *Firmicutes* expressing extracellular α-(1 → 4)-glucan branching enzyme (GH13) and *Bacteroidetes* expressing periplasmic neopullulanase (GH13) and α-glucosidase (GH97). Fructans were degraded by extracellular GH32 enzymes from *Bacteriodetes* and *Lactobacillus*. Lactose fermentation by β-galactosidases (GH2 and GH42) was identified in *Firmicutes*. In conclusion, the assembly of 360 high-quality genomes as the first metagenomic reference for swine intestinal microbiota allowed identification of key microbial contributors to degradation of starch, fructans, and lactose.

**Conclusions:**

Microbial consortia that are responsible for degradation of these glycans differ substantially from the microbial consortia that degrade the same glycans in humans. Our study thus enables improvement of feeding models with higher feed efficiency and better pathogen control for weanling pigs.

**Electronic supplementary material:**

The online version of this article (10.1186/s40168-019-0662-1) contains supplementary material, which is available to authorized users.

## Introduction

Culture-dependent and culture-independent approaches have advanced our understanding of the assembly of intestinal microbiota and their importance for their host [1–4]. In general, the development of gut microbiota is influenced by host genetic variation [2, 5, 6], environmental factors, and stochastic events [5, 7, 8]. The association between the host genetics and gut microbiome is mediated by immunity-related pathways and the secretion of antimicrobial compounds [2]. Multiple environmental factors, such as antibiotics, social contacts, and the environment, also shape the architecture of gut microbiota [5, 7, 9]. The diet and particularly dietary carbohydrates are key determinants for the composition and activity of the intestinal microbiome [1, 5, 7, 10, 11].

At weaning, mammals gradually transition from lactose to plant carbohydrates as main source of dietary carbohydrates; this dietary shift also induces a major shift of the intestinal microbiome. In contrast, current swine production systems impose an abrupt transition from sow’s milk to solid food in piglets; this also induces an accelerated succession of microbial communities in weanling piglets [7, 12]. Gut microbial communities play a pivotal role in facilitating adaption of weanling piglets to fibrous feed and in minimizing the risk of colonization by pathogens after weaning [13]. Carbohydrates are the main energy source for pigs; in commercial pig production, carbohydrates account for more than 60% of the dry matter and 60–70% of the dietary energy intake [14, 15]. However, digestive enzymes secreted by the host do not degrade dietary polysaccharides other than starch. Pigs and other mammalian hosts rely on symbiotic gut microorganisms to ferment this abundance feed ingredient and provide metabolic energy [8]. Because specific microbial taxa are specialized for degradation of specific dietary carbohydrates, the composition of the diet alters the composition and activity of intestinal microbiota [15].

Current analyses of intestinal microbiota are largely based on sequence-based methodology, avoiding the time-consuming culture-based analysis of intestinal microbiota. When assessing the function of intestinal microbiota on the basis of high-throughput sequencing data, metagenomic binning and genome-scale metabolic reconstructions has bridged the gap between the taxonomic analysis of microbial communities on the basis of 16S rRNA sequences and the description of the metabolic repertoire of individual members of gut microbiome by analysis of the abundance and distribution of metabolic enzymes [16]. Metagenomic binning is also an essential tool to understand metabolic cooperativity between different representatives of the microbiome. Metabolic binning of metagenomic sequence data and the assignment of bacterial taxonomy to metabolic activity is thus an important tool to substitute untargeted microbiome modulation with targeted or predictable modulation of gut microbiome [17].

Swine are an important livestock species. Particularly at weaning, dietary management of the microbiome of piglets is an important tool to reduce the susceptibility to pathogens, and to improve feed efficiency. However, past studies of the interactions between gut microbiome and diets in pigs were limited to 16S rRNA gene sequencing, or metagenomic analyses without metagenomic binning [7, 18–20]. This study therefore aimed to unravel the adaptation of the swine microbiome to the dietary shift after weaning, and to establish a metagenomic reference by binning of genomes of swine gut bacteria from 72 samples from 18 animals. The metagenomic reference was used to predict the metabolic capacity of the fecal microbiome for metabolism of dietary carbohydrates by CAZy annotation, and by detailed analysis of metabolic pathways of major substrates present in wheat.

## Materials and methods

### Diets, animals, and samples

Six experimental diets were prepared by mixing 98% basal diet (Additional file 1: Table S1) with 2% unfermented wheat, acidified wheat (pH 3.8), wheat fermented with *Lactobacillus casei* K9-1 and *Lactobacillus fermentum* K9-2 (CanBiocin Inc., Edmonton, AB, Canada), or unfermented wheat with freeze-dried cultures of *L*. *casei* K9–1 and *L*. *fermentum* K9-2 (approximately 10^9^ CFU /g), wheat fermented with *Lactobacillus reuteri* TMW1.656, and wheat fermented *L*. *reuteri* TMW1.656Δ*rtcN* (Additional file 1: Table S2) [21]. Feed fermentation was performed as previously described [12, 20].

The six dietary treatments were randomly allocated to 48 crossbred castrated male piglets (21 days of age) with randomized block design to provide 8 replicates per dietary treatment. Pigs were raised in a temperature-controlled room (28 ± 2 .5 °C) with one pig per pen and divided into six blocks. Pigs had access to ad libitum feed and clean water.

A total of 191 fecal samples were collected from the pen floors at days at weaning (0 day) and 7, 14, and 21 days after weaning. To avoid contamination of fecal samples, the pen floor was cleaned before sample collection and samples were collected during the peak hours of defecation 1–2 h after morning feeding. Fecal samples were immediately stored at − 20 °C after sampling. Subsampled samples (2–3 g) were stored at − 80 °C after thawing and mixing of frozen samples. Total bacterial DNA was extracted from fecal samples using QIAamp Fast DNA stool mini kit (Qiagen, Inc., Valencia, CA, USA) pre-treated with bead-beating (BioSpec Products, Inc., Bartlesville, USA) for 30 s × 8 times. Purified DNA with an A260/280 ratio higher than 1.8 were selected for sequencing analysis. PicoGreen quantification assay was included by default as quality control for 16S rRNA gene sequencing (University of Minnesota Genomics Center, Minneapolis, MN, USA) and shotgun metagenomic sequencing (McGill University and Genome Quebec Innovation Center, Montreal, Canada).

### Intestinal microbial community analysis using 16S rRNA gene sequencing

Genomic DNAs were sequenced on Illumina MiSeq (2 × 300 bp reads) by amplifying the V5-V6 domain of the 16S rRNA gene using TruSeq Nano protocol (University of Minnesota Genomics Center, Minneapolis, MN, USA). A total of 6,647,893 sequences with an average length of 266 bp, corresponding to 34,805 16S rRNA sequences for each of the 191 samples, were retained for downstream analysis after the quality filtering from QIIME pipeline (MacQIIME 1.9.1) [22]. Sequences with 97% similarity were clustered into operational taxonomic units (OTUs) by UCLUST [23] after de-replication and de-multiplexing. The GreenGenes database was used for taxonomy assignment with the default cut-off of 97% average nucleotide identity (ANI) at the genus level [24]. OTUs that were represented by only one or two sequences (relative abundance < 0.005%) were discarded. Principle coordinates analysis (PCoA) and analysis of similarities (ANOSIM) were performed using weighted UniFrac distance matrix calculated by *beta*_*diversity.py* [7].

### Metagenomic sequencing, assembly, binning, and genome annotation

Samples from 18 pigs were randomly selected to include 6 samples from each of the following dietary groups: acidified wheat, wheat fermented with *Lactobacillus casei* K9-1 and *L*. *fermentum* K9-2, or unfermented wheat with freeze-dried cultures of *L*. *casei* K9-1 and *L*. *fermentum* K9-2. Feces taken from these 18 piglets at weaning (0 day) and 7, 14, and 21 days after weaning were sequenced on Illumina Hiseq 2500 PE125 platform with low input shotgun metagenomic library protocol (McGill University and Genome Quebec Innovation Center, Montreal, Canada). A total of 399.25 Gb of sequence data were obtained, corresponding to 2.22 × 10^11^ reads for each of the 72 samples. After quality check by FastQC, adapters were trimmed from raw reads by Trimmomatic [25] using a local adapter database. Trimmed reads were assembled into contigs using IDBA_UD with default parameters.

Binning was performed with MaxBin2 [26] using contigs longer than 3000 bp. After a two-step de-replication with dRep [27], 596 bins were obtained from the sample clusters pooled by pigs (four time points for each pig). CheckM [28] assessment indicated that all 596 bins were ≥ 50% complete; of these 596 bins, 458 bins were substantially complete (completeness ≥ 70%) and 240 bins were nearly complete (completeness ≥ 90%) [29] (Additional file 1: Table S3). Of the 458 substantially complete bins, 360 bins with contamination < 5% were regarded as high-quality assembled genomes and selected for further analyses.

Open reading frames (ORFs) were identified by prodigal v.2.6.1 [30]. ORFs were annotated with BLAST against Clusters of Orthologous Groups (COG) database and CAZy database with an *e* value ≤ 1e-5 [31].

### Phylogenetic identification and calculation of the relative abundance

The taxonomy of 360 high-quality bins was assigned by *Phylophlan* with 3737 reference genomes [32] on the basis of 400 proteins. The bins were assigned at the species, genus, and family level when average amino acid identity of encoded proteins to the reference genome was greater than ≥ 90%, 60%, and 45%, respectively [33, 34], in at least 50 proteins [35]. The average coverage of bins were determined using MaxBin2 [26] by recruiting reads (from each sample) to scaffolds. The average coverage normalized to the total number of reads in each sample corresponds to the relative abundances of bins.

### Reconstruction of metabolic pathways for carbohydrate fermentation

CAZy were clustered into five categories based on the substrate specificity of glycoside hydrolases (GHs) and carbohydrate esterases (CEs). Enzymes from the GH families GH13, GH31, GH97, GH4, GH14, GH15, GH57, and GH63 were assigned to starch-degrading enzymes. GH families GH32, GH91, and GH68 were assigned as fructan-hydrolyzing enzymes. GH families containing β-glucanases including licheninase, β-glucan endohydrolase, endo-(1, 4) β-glucanase are GH8, GH16, GH26, GH5, GH6, GH9, GH10, GH12, GH44, GH48, GH45, GH51. GH and CE families harboring xylanase, arabinofuranosidase, α-glucuronsidase, and acetyl-xylan esterase were regarded as arabinoxylan-specific and include GH5, GH10, GH11, GH8, GH43, GH51, GH67, GH115, CE1, CE2, CE4, CE6, and CE7. Enzymes degrading O-linked and N-linked host glycans include GH20, GH84, GH110, GH89, GH125, GH109, CE14, GH123, and CE9. The degradation capacity of each bin corresponds to the sum of positive hits of GHs or CEs under each category.

Metabolic pathways of starch, fructan, and lactose were studied by blasting sequences of key enzymes that were characterized biochemically (Additional file 1: Table S4) against 360 assembled genomes. An amino acid identity of ≥ 40% and *e* value ≤ 1e-5 were used as threshold values. The relative abundance of enzymes over time was calculated by sum of all positive hits normalizing with corresponding abundance of target bins at four time points.

### Statistical analysis

The data for average daily gain, feed intake of pigs, feed efficiency, and relative abundance of bins were analyzed using linear mixed-effects (LME) models in R (version 3.4.3). In the model, time was treated as fixed factor; pig was considered as experimental unit and random factor. *P* values < 0.05 with Bonferroni-adjustment were considered significant. Results are presented as means ± standard deviation. Alpha-diversity parameters between time points were compared using Kruskal-Wallis rank-sum test. Two-way ANOVA was applied to investigate the longitudinal differences of UniFrac distances between littermates and between all pigs from different litters. Statistical significance of ANOSIM was determined through permutations between dietary categories. The *R* value was calculated by the following formula: *R* = difference of mean rank (all distances between groups − all distances within groups) / (N(N-1)/4). The larger *R* value between 0 and 1 reflects the higher dissimilarity between the groups.

## Results

### Growth performance and gut health of pigs

The grow performance of pigs in 21 days after weanling is listed in Table 1. Both average feed intake and average daily gain increased throughout the experimental period. The feed efficiency increased in the first 2 weeks and plateaued in the last week. All animal remained healthy during the 21-day trial.

**Table 1:** Growth performance of weanling pigs during the first 3 weeks after weanling

Time	Day 7	Day 14	Day 21
Average feed intake^a^ (g DM^b^/day)	266.56 ± 2.90 ^C^	468.98 ± 4.11 ^B^	749.07 ± 16.85 ^A^
Average daily gain (g/day)	177.31 ± 11.48 ^C^	375.00 ± 11.33 ^B^	615.38 ± 17.49 ^A^
Feed efficiency (G/F)	0.65 ± 0.04 ^B^	0.80 ± 0.02 ^A^	0.84 ± 0.03 ^A^

Data was presented as mean ± standard error of means. Results with unlike letter in the same row were significant different (*P* < 0.05)

^a^Pigs were fed with phase 1 diet (80% basal diet + 20% wheat flour) for the first 7 days, followed by phase 2 diet (50% basal diet + 50% wheat flour) from days 8 to 21

^b^*DM* dry matter

### Bacterial community composition analysis by 16S rRNA gene sequencing

**Fig. 1:**
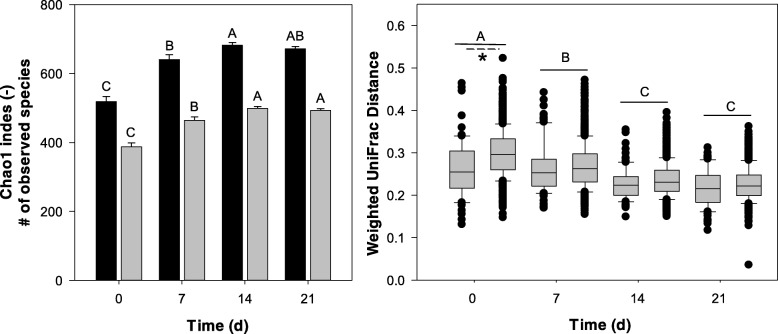
**a** α-Diversity of fecal microbiota over time. Black bars represent Chao1 indexes, gray bars represent the number of observed species in each sample. Data were calculated from partial 16S rRNA sequences and are presented as mean ± standard errors of the means (*n* = 48). Mean values for the same index (bars with same color) with unlike letters or asterisk (*) are significantly different (*P* < 0.05). **b** UniFrac distance (weighted) between fecal microbiota of piglets from the same sow (gray bars) and from all piglets (hatched bars) during the first 3 weeks after weanling. Mean values for the same group (bars with same color) and pairs at the same time point with unlike letters are significantly different (*P* < 0.05)

Analyses of the microbiome composition determined factors that influence the evolution of the microbiome after weaning (Fig. 1 and Additional file 1: Figure S1). Alpha diversity increased after weaning and remained stable after week 3 (Fig. 1). The presence of probiotic lactobacilli in the diet did not influence the composition of fecal microbiota. Significant but minor differences were observed between individual animals (Table 2). Litter effects were significant at weaning but not at later sampling times (Fig. 1). The differences between bacterial communities were mainly explained by wheat content of the diet and the time after weaning (Table 2). The effects of time and wheat inclusion on bacterial composition were visualized by principal coordinates plots (PCoA) based on weighted UniFrac distance matrix (Additional file 1: Figure S1). PCoA clearly grouped samples based on time after weaning and wheat content (Additional file 1: Figure S1). Therefore, subsequent analyses focused on microbial degradation of carbohydrates.

**Table 2:** ANOSIM of fecal microbiota after weaning based on weighted UniFrac distance matrix calculated with partial 16S rRNA sequences

Factors	ANOSIM parameters		
	*N*	*R* ^a^	*p*
Probiotic	6	0.008	0.122
Animal	48	0.087	0.001
Sow	11	0.100	0.001
Age	4	0.332	0.001
Wheat	3	0.505	0.001

*ANOSIM* analysis of similarity

^a^Slight correlation was considered when 0 < *R* < 0.3, whereas *R* > 0.3 was considered a strong correlation

### Metagenomic reconstruction of fecal microbiome in weanling pigs

**Fig. 2:**
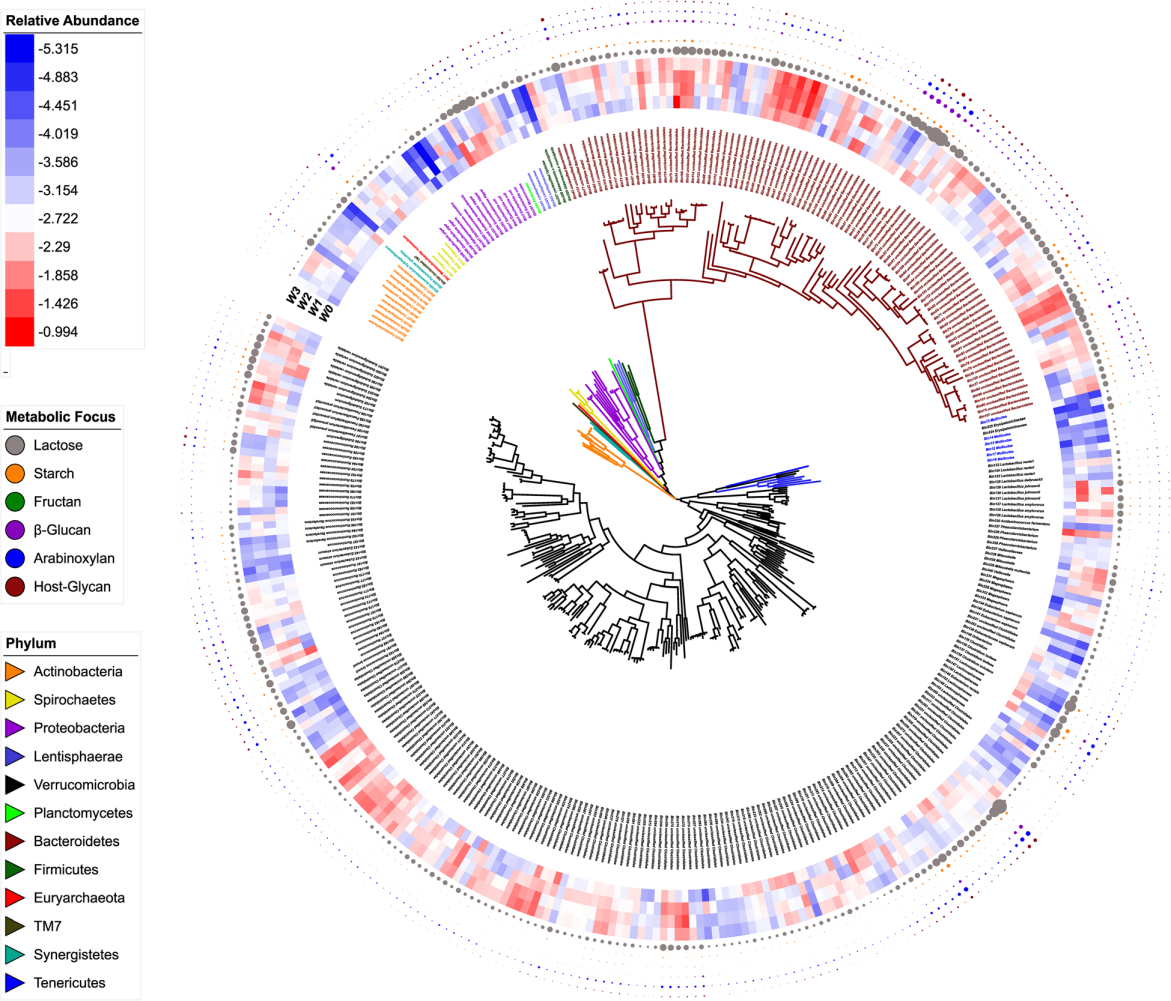
Phylogeny, abundance, and metabolic potential of bacterial taxa in the fecal microbiota of piglets. Bacterial taxa were identified based on reconstructed genomes assigned to 360 bins with ≥ 70% completeness and < 5% contamination. The phylogenetic tree and the taxonomic assignment of reconstructed bins are shown as the innermost layers. The taxonomic assignment was based on the average amino acid identity of encoded proteins to the most closely related reference genome sequence. Branches and labels with different colors represent different phyla as indicated by the color code to the lower left. The heatmap in the third layer depicts the relative abundance of the 360 bins, inside to outside 0, 7, 14, and 21 d (*n* = 18 per time point). The relative abundance of bins in each sample was calculated from the average contig coverage obtained by re-mapping reads form samples and normalizing to the total reads in the sample. The outermost four layers depict the number of glycosyl hydrolases and esterases encoded in each bin. Glycosyl hydrolases and esterases were grouped by their predicted substrate specificity as follows: Lactose-degrading enzymes include GH1,GH2, and GH42; starch-degrading enzymes include GH13, GH31, GH97, GH4, GH14, GH15, GH57, and GH63; fructan-degrading enzymes include GH32, GH91, and GH68; β-glucan-degrading enzymes include GH8, GH16, GH26, GH5, GH6, GH9, GH10, GH12, GH44, GH48, GH45, and GH51; arabinoxylan-degrading enzymes include GH5, GH10, GH11, GH8, GH43, GH51, GH67, GH115, CE1, CE2, CE4, CE6, and CE7; host-glycan-degrading enzymes include GH20, GH84, GH110, GH89, GH125, GH109, CE14, GH123, and CE9

Reconstruction of bacterial genomes from metagenomic sequence data generated a total of 596 genomic bins from 18 weanling pigs and enabled a genome-based investigation of microbial metabolism. The average size of 596 bins was 2.07 Mb and the average length of N50 was 32,152 bp. Figure 2 shows the taxonomic identification of 360 bins with completeness of > 70% and contamination of < 5%. Of the 360 identified bins, 216 were assigned to *Firmicutes* and 96 to *Bacteroidetes*; 11 bins were identified as *Actinobacteria*, 16 as *Proteobacteria*. Only 106 of the metagenomics bins were identified at the genus or species level; remaining bins did not match to genome-sequenced reference strains. The relative abundance of bacterial genomes was calculated based on the average coverage per metagenomics bin normalized to the number of total reads in each sample (Fig. 2, Additional file 1: Table S3). About half of the bins (175 of 360) showed differences in abundance over time. Among these, 56 bins showed a higher abundance at 0 day when compared to other sampling times while the abundance of 64 bins increased over time. One of the bins with decreasing abundance represents *Lactobacillus delbrueckii*, which has to date not been considered a representative of animal intestinal microbiota. Interestingly, 20 bins only increased temporarily at day 7 and/or day 14.

The capacity of 360 bins for glycan degradation was initially predicted by identification of glycoside hydrolases (GHs) and carbohydrate esterases with similar substrate preference (Fig. 2, Additional file 1: Table S3). Starch-degrading enzymes were widely found in genomes of *Firmicutes* and *Bacteroidetes*. These metagenomic bins were relatively abundant and increased over time, particularly in genomes of *Faecalibacterium* spp. Only few genomes harbored fructan-degrading enzymes, examples include genomes of *Lactobacillus*, *Escherichia coli*, and several unclassified *Bacteroidales*. The distribution of bins carrying enzymes for β-glucan, arabinoxylan, and host-glycan metabolism overlapped; these GH families were widely distributed in genomes of *Bacteroidetes*, *Ruminococcus*, and *Lachnospiraceae*.

### Reconstruction of metabolic pathways for starch, fructan, and lactose metabolism in weanling pigs

**Fig. 3:**
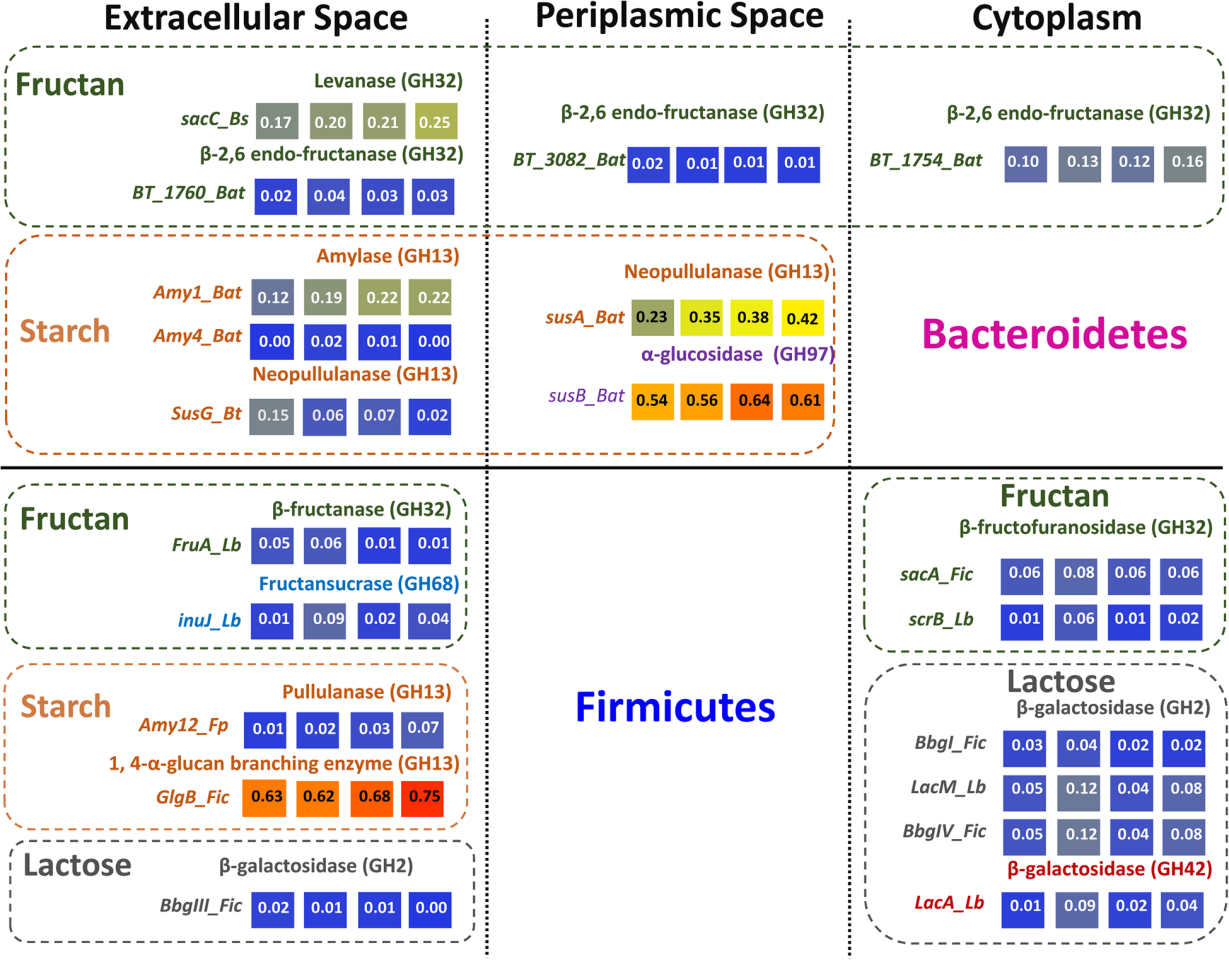
Predicted metabolic pathways for starch, fructan, and lactose metabolism. The abundance of metabolic enzymes was obtained by using biochemically characterized enzymes as query sequences for BLAST analysis of metagenomics bins. Enzymes are grouped by the substrate and the cellular location of the query sequence. The abundance of corresponding genes at the four time points was calculated from the cumulative relative abundance of bins encoding for a homolog of the gene, and is shown as color coded matrix for the four time points (left to right 0, 7, 14, and 21 d). Labels at the left side for rows include the name of gene and the abbreviations for the organism for which the corresponding enzyme was characterized. Abbreviations for organisms are as follows: *Bat Bacteroidetes*, *Bs, Bacillus subtilis*,  *Bt Bacteroides thetaiotaomicron*, *Lb Lactobacillus*, *Fp Faecalibacterium prausnitzii*, *Fic Firmicutes.* The accession number of query sequences and reference to the biochemical characterization of the enzymes is provided in Additional file 1: Table S4 of the online supplementary material

Microbial metabolism of starch, fructan, and lactose was further analyzed by identification of metabolic enzymes degrading these carbohydrates [36–43] (Fig. 3). Query sequences were selected to retrieve all characterized metabolic pathways for the substrate with minimal overlap between hits obtained with different query sequences for the same substrate.

*Firmicutes* and *Bacteroidetes* harbored distinct pathways for starch degradation. *Firmicutes* convert starch by an extracellular α-(1 → 4)-glucan branching enzyme (GlgB) and pullulanases (Amy12); these enzymes occurred only in *Firmicutes.* GlgB was detected in 191 of 216 bins assigned to *Firmicutes*. Remarkably, most of the starch-degrading *Firmicutes* carry GlgB but no other starch-hydrolyzing enzymes; only five members of *Faecalibacterium prausnitzii* and one *Eubacterium rectale* additional carry pullulanases. Bin 254 was the only member of *Firmicutes* with an extracellular neopullulanase2. The periplasmic starch utilization by *susA* (GH13, 54.2%) and *susB* (GH97, 74.0%) was present in *Bacteroidetes*. Multiple metagenomics bins assigned to *Bacteroidetes* contained multiple genes for starch digestion, including extracellular and periplasmic enzymes. Among the 80 starch-degrading *Bacteroidetes*, only 2 *Bacteroidetes* were without *susA* or *susB*, whereas 23 *Bacteroidetes* additionally harbored extracellular amylase (Amy1, 4) or neopullulanase (SusG).

*Firmicutes* and *Bacteroidetes* also harbored distinct enzymes for fructan hydrolysis. *Firmicutes* catabolize fructan by intracellular β-fructofuranosidase (ScrA/ScrB, GH32) and extracellular fructansucrases (Inu, GH68, and FruA, GH32). FruA, ScrB, and Inu were present only in lactobacilli. ScrA was present in other *Firmicutes*, including *Subdoligranulum variabile*, *Faecalibacterium prausnitzii*, *Eubacterium*, and unclassified *Clostridiales*. *Bacteroidetes* metabolize fructan by β-(2 → 6) endo-fructanases (GH32) including BT_1760 (extracellular), BT_3082 (periplasmic), and BT_1765/1754 (intracellular). Among 49 fructan-degrading *Bacteroidetes*, only three members did not carry intracellular β-(2 → 6) endo-fructanase, nine members of *Bacteroidetes* additional harbored extracellular BT_1760, another two members additional carried periplasmic BT_3082.

Lactose hydrolysis was identified only in *Firmicutes* with exception of bin20 representing a member of *Coriobacteriaceae*. Most lactose-degrading bacteria (27 out of 31) hydrolyze lactose by intracellular GH2 β-galactosidase (BbgI, LacM, and BbgIV) or GH42 β-galactosidase LacA, including members of *Lactobacillus*, *Subdoligranulum*, and *Ruminococcus*. The abundance of *Lactobacillus delbrueckii*, a species that is specialized for lactose conversion, decreased over time (Fig. 2). The other lactose decomposers were capable of lactose hydrolysis by extracellular BbgIII (GH2).

## Discussion

### Microbial composition in piglets differed by host-derived factors and reshaped by diet

The fecal microbiota of weanling pigs is unstable during early life and stabilizes within 2–3 weeks after weaning [12, 18]. In this study, we analyzed the structure and function of the fecal microbial community in piglet during the first 3 weeks after weaning by partial 16S rRNA gene sequencing and metagenomics analysis. Analysis of fecal microbiota enables sampling from the same animal over time. Swine fecal microbiota closely resemble microbiota of the colon, where complex carbohydrates are fermented, but differ substantially from ileal microbiota that are specialized on fermentation of mono- and disaccharides [43–46].

Diet together with age were the most significant factors shaping community assembly in weanling piglets; litter effects were transient and minor. Age alters the physiology of the gastrointestinal tract including immune and metabolic functions during weaning in piglets; these changes occur rapidly in response to the transition to solid food [47, 48]. Initial differences in microbiota structure between animals and litters were altered by the uniform post-weaning diet. Inclusion of probiotic bacteria modulated gastric communities of lactobacilli but did not significantly alter the structure of fecal microbiota [21]. The use of the same probiotic *L*. *reuteri* at a tenfold higher dose significantly altered the abundance of only very few bacterial taxa [19]. The limited impact of probiotic bacteria on the overall composition of intestinal microbiota matches observations in humans [47, 48]. Most lactobacilli used in probiotic studies are allochthonous to the hosts’ gastrointestinal tract. These allochthones are less likely to alter autochthonous microbiota than host-adapted probiotic strains [21, 48, 49].

### Novel reference with 596 genomes were reconstructed for swine fecal microorganisms

The importance of the intestinal microbiome for host physiology highlights the need for comprehensive analysis based on genomic and phenotypic assays. Culture-dependent analysis of intestinal microbiota, however, lags the identification of bacterial taxa by high-throughput sequencing approaches [3, 50]. This study reconstructed 596 genomes including 360 high-quality and substantially completed genomes from swine fecal microbiota. Owing to the high error rates in sequencing and assembly of regions with repetitive sequences including rRNA operons [51], bacterial taxa were identified on the basis of the average amino acid identity (AAI) of multiple conserved proteins sequences distributed across the genomes [33]. About 30% of the genomes—106 of 360 genomes—were assigned to the genus or species level. This greatly expands the taxonomic assignment of swine microbiota when compared to previous metagenomic studies conducted without metagenomic binning, which assigned 7.6% of taxa to the species or genus level [20], but also emphasizes the need for further metagenomic and culture-based studies to characterize previously unidentified bacterial taxa. For the remaining 254 high-quality genomes in this study, we provide novel genome sequence data and expand current knowledge on metabolic diversity of swine intestinal microorganisms [52, 53]. The phylogenetic analysis of 360 reconstructed genomes and the annotation of open reading frames serves as the first reference for metagenomes of swine microbiota. Different from past swine metagenomics studies [7, 18, 54], this reference allows the assignment of the metabolic activity of intestinal organisms to their taxonomic identification.

### Phylogeny and functions of high-quality genomes reconstructed for swine fecal microorganisms

The increasing abundance of *Bacteroidales* and *Clostridiales* over time reflects their ability to derive metabolic energy from diverse plant polysaccharides [41, 55–57]. CAZy annotation indicated the ability of multiple *Bacteroidetes* species to degrade starch, β-glucan, arabinoxylan, and host glycans [37]. *Ruminococcaceae*, a family in the phylum *Firmicutes*, also includes species with the capability to hydrolyze a broad range of polysaccharides, matching the identification of *Ruminococcus bromii* as a keystone species for starch degradation in the human colon [58]. The relative abundance of metagenomic bins representing bacterial taxa with multiple polysaccharide-degrading enzymes increased in response to the inclusion of plant carbohydrates after weaning. Examples include members of *Bacteroidetes* and *Ruminococcaceae*. In contrast, some early colonizers, mostly *Proteobacteria* including *E*. *coli* and members of the genus *Clostridium*, decreased dramatically after weaning. The reduced abundance of *Proteobacteria* may relate to a lower protein intake concomitant with a higher fiber intake and a more developed immune function [47, 59].

The relative abundance of the species *Lactobacillus amylovorus*, *Lactobacillus johnsonii*, and *Lactobacillus reuteri* that was determined by metagenomic binning (this study) matched previous analyses of the same samples that were based on 16S rRNA sequencing and species-specific quantitative PCR [21]. Metagenomic binning also identified *Lactobacillus delbrueckii* in the microbiome of piglets; this organism decreased rapidly after weaning. *L*. *delbrueckii* has not been considered a member of animal intestinal microbiota [60]. Different from other species of the *L*. *delbrueckii* group, which maintain enzymes for metabolism of a relatively broad array of carbohydrates, the genome of *L*. *delbrueckii* underwent reductive evolution that silenced most carbohydrate metabolic enzymes [61]. This metabolic focus of *L*. *delbrueckii* on lactose as main source of metabolic energy was interpreted as adaptation to the milk environment or dairy fermentations [61]; however, our data suggests that the metabolic focus on lactose may alternatively represent adaptation to the intestine of suckling mammals. Re-analysis of the intestinal microbiome of weanling piglets [19] indeed revealed that *L*. *delbrueckii* was also detected in piglets on the day of weaning but no longer detectable 2 or 3 weeks after weaning (Additional file 1: Figure S2).

### Microbial degradation of starch, fructans, and lactose in weanling pigs

Even though a large panel of CAZymes have been cataloged based on substrate specificity, CAZy family-based classification of enzymes needs to be complemented by a more detailed analysis that is based on reference sequences of enzymes that were biochemically characterized [37, 62]. Moreover, classification of proteins in GH or CE families not always allows an unambiguous prediction of their substrate specificity or cellular location. Many substrates are degraded by enzymes from several families, and enzymes in many GH or CE families are active on more than one substrate [55].

In humans, starch entering the large intestine is degraded by microbial consortia contributing diverse extracellular, periplasmatic, and intracellular starch-converting and -hydrolyzing enzymes [63] while lactose, GOS, and dietary fructans are degraded by few bacterial groups, particularly *Bifidobacterium* spp. [64, 65]. Our analysis revealed that microbial consortia and species that degrade starch, fructans, and lactose in weanling piglets differ substantially from microorganisms or microbial consortia that are responsible for the corresponding metabolic activities in human intestinal microbiota.

Bacterial degradation of starch is mediated by amylases and pullulanases, which hydrolyze α-(1 → 4)- and α-(1 → 6)-glucosidic bonds, respectively [39]. Members from GH13 families were identified as the principal starch degrading enzymes but *Firmicutes* and *Bacteroidetes* used distinct starch-utilization systems*.* Extracellular glycosidases were identified mainly in *Firmicutes* while periplasmic enzymes were only found in *Bacteriodetes*. The high abundance of an extracellular α-(1 → 4)-glucan branching enzyme suggests this enzyme is important for the primary degradation of starch in the swine GIT. The α-(1 → 4)-glucanotransferase GlgB catalyzes glucan chain transfer to form α-(1 → 6)-glucosidic linkages; this enzyme was found in *Firmicutes* only. The enzyme was suggested to improve accessibility to insoluble starch [40, 66] and is broadly distributed in intestinal microbiota of different hosts including humans, chicken, cattle, and swine [67]. Following starch hydrolysis by extracellular enzymes, the α-glucosidases SusA and SusB further degrade gluco-oligosaccharides in the periplasm. Disruption of SusA and SusB from *Bacteroides thetaiotaomicron* reduced the rate of growth but did not eliminate the growth of the strain [68]; however, periplasmic starch-degrading enzymes may reduce access of competitors to the products of hydrolysis. With exception of *F*. *prausnitzii*, starch-degrading *Firmicutes* carried a single glycosidase; in contrast, redundant enzymes with different activities or different locations were commonly detected within a single genome of *Bacteroidetes*. The high abundance of starch-utilizing enzymes in fecal microbiota demonstrates that wheat starch, despite its hydrolysis by pancreatic amylases and brush border enzymes, is a major carbohydrate source for colonic microbiota. The distribution of extracellular and periplasmic enzymes for starch degradation highlights a high level of metabolic cooperativity that was also noted in human starch and cellulose-degrading microbial communities [37, 58]. In humans, *Ruminococcus bromii* plays a key role in fermentation of type 3-resistant starch and enhanced the growth of *B. thetaiotaomicron*, *Bacteroides adolescentis*, or *E*. *rectale* on resistant starch [42, 58]; however, this study indicates that this species does not fulfill a comparable role in swine microbiota.

Low molecular weight fructans are among the major non-starch polysaccharides in wheat [69]. Fructans were degraded by *Bacteriodetes* and lactobacilli*.* The linear structure of fructans allows hydrolysis by single enzyme that is classified in the GH32 or GH68 families [40]. In contrast to the complex and partially redundant starch-degrading enzymes in *Bacteriodetes*, fructans degraders carried fewer fructanases. Fructan utilization is not conserved within members of a specific species [41, 42, 70]. Three GH32 enzymes, including BT1760 (extracellular), BT3082 (periplasmic), and BT1765 (intracellular), as well as hybrid two-component (HTC) signaling system, BT1754 are required for fructan utilization in *B*. *thetaiotaomicron* and related *Bacteroides* spp. [41]. *B*. *thetaiotaomicron* utilized levan while *Bacteroides caccae* ferments inulin [41, 69]. GH68 family enzymes in lactobacilli are extracellular levansucrases which are necessary for biofilm formation on non-secretary epithelia of the upper GI tract; these enzymes synthesize levan but do not contribute to fructan hydrolysis [71]. Intracellular GH32 β-fructofuranosidases of lactobacilli (ScrB) utilize only di- and trisaccharides that are transported across the membrane [36, 70, 72]. The metagenomic analysis is the first to report the presence of extracellular fructanases (FruA) in intestinal lactobacilli. FruA is common in oral streptococci; however, its presence is exceptional in lactobacilli and was previously identified only in type II sourdough microbiota [43]. The exclusive presence of FruA in *Lactobacillus* species representing swine intestinal communities may reflect specific nutritional requirements in pigs. The identification of *Bacteroides* and *Lactobacillus* spp. as major fructan-degrading organisms also differentiates human and swine microbiota; in humans, bifidobacteria are the main organisms that degrade fructans [73].

Lactose accounts for about 26.7% of sow milk solids [74]; transition diets contain 10–15% of lactose. Lactose is a major dietary carbohydrate in suckling and weanling pigs. Only *Firmicutes* fermented lactose with the LacS/LacLM pathway widely distributed in lactobacilli [75–77]. Lactose is transported into the cytoplasm by lactose permease and hydrolyzed by intracellular GH2 β-galactosidases common in *Firmicutes*, and GH42 β-galactosidases of lactobacilli. Lactobacilli colonize the stomach of swine where dietary lactose is available; in contrast, *Bacteroidetes* are dominant only in hindgut microbiota after full or partial digestion of lactose in the small intestine. Accordingly, their extracellular enzymes may target β-glycosidic linkages in host or plant glycans rather than lactose. Microbial fermentation of lactose in the terminal ileum and the large intestine contributes to lactose digestion particularly in lactase-non-persistent humans [78]. The distribution of β-galactosidases in human microbiota remains poorly characterized; bifidobacteria are considered to be the main organisms involved in metabolism of lactose and related β-galacto-oligosaccharides [79].

In conclusion, we present a metagenomic reference for swine fecal microbiome by assigning taxonomies and metabolic functions to the 360 high-quality assembled genomes. Along with the clear evidence for dietary carbohydrates acting as the most significant drivers for diversification of microbiota, we further determined the key microbial contributors to degradation of major substrates in starter diet, including starch, fructans, and lactose. Starch is a substrate for colonic microbiota and its metabolism is dependent on metabolic cooperativity between *Firmicutes* and Bacteroidetes. Fructans and lactose are fermented by simple enzyme systems present in *Bacteroides* and *Lactobacillus* spp., respectively. Our genome-based functional analysis thus improves the understanding of carbohydrate fermentation in the swine intestine when compared to previous studies that report gene annotation without metagenomic binning [20, 44]. It will enable future studies linking composition and function of piglet microbiota to establish feeding systems that improve feed efficiency and animal health while reducing microbial resistance to antibiotics. It may also facilitate the design and interpretation of swine as a highly suitable animal model to understand carbohydrate digestion in the human intestine [51, 79–81].

## Additional file

Additional file 1: **Table S1.** Ingredient composition of basal diets. **Table S2.** Experimental design and diets. **Figure S1.** Principle coordinates analysis (PCoA) of fecal microbiota composition. **Table S3.** Quality assessment of 596 bins by CheckM. **Table S4.** Accession numbers of enzymes blast for starch, fructan and lactose degradation. **Figure S2.** Relative abundance (%) of *Lactobacillus delbrueckii* of suckling pigs (day 0) and weaned pigs (day 7 and day 14). Data were determined by Illumina sequencing of 16S rRNA tags in a previous experiment (19). Data with unlike letters are significantly different (*P* < 0.05). (PDF 1186 kb)
