# Supplementary material for: Metagenomic reconstructions of gut microbial metabolism in weanling pigs
Source: Microbiome. 2019 Mar 26;7:48. doi: 10.1186/s40168-019-0662-1 (PMC6436221; doi:10.1186/s40168-019-0662-1)
Supplement: Supplementary file 1 — Table S1. Ingredient composition of basal diets. Table S2. Experimental design and diets. Figure S1. Principle coordinates analysis (PCoA) of fecal microbiota composition. Table S3. Quality assessment of 596 bins by CheckM. Table S4. Accession numbers of enzymes blast for starch, fructan and lactose degradation. Figure S2. Relative abundance (%) of Lactobacillus delbrueckii of suckling pigs (day 0) and weaned pigs (day 7 and day 14). Data were determined by Illumina sequencing of 16S rRNA tags in a previous experiment (19). Data with unlike letters are significantly different (P < 0.05). (PDF 1186 kb) [file 40168_2019_662_MOESM1_ESM.pdf]

Online supplementary material

Metagenomic Reconstructions of Gut Microbial Metabolism in Weanling Pigs

Table S1. Ingredient composition of basal diets

Table S2. Experimental design and diets

Figure S1. Principle coordinates analysis (PCoA) of fecal microbiota composition

Table S3. Quality assessment of 596 bins by CheckM

Table S4. Accession numbers of enzymes blast for starch, fructan and lactose degradation

Figure S2. Relative abundance (%) of *Lactobacillus delbrueckii* of suckling pigs (day 0) and weaned pigs (day 7 and day 14). Data were determined by Illumina sequencing of 16S rRNA tags in a previous experiment (19). Data with unlike letters are significantly different ($P < 0.05$)

Table S1. Composition of diets to fulfil NRC recommendations (2012) for pigs (5-11kg).

Ingredients (%)	Phase 1 (day 0 to 6)	Phase 2 (day 7 to 21)
Wheat HRS (NRC)	20.00	50.00
Corn (NRC)	31.54	1.76
Lactose	15.00	10.00
Soybean meal (NRC)	15.00	15.00
B. napus canola meal		2.50
Soy protein conc HP300	3.00	2.50
Herring meal	6.00	2.50
Corn DDGS [a] (NRC)		5.00
Canola oil (NRC)	4.00	3.40
Limestone	1.15	1.10
Salt	0.50	0.50
Other vitamin and mineral ingredients	3.31	5.24
TiO2	0.50	0.50
Total	100	100

[a] DDGS, distiller’s dried grains with soluble

Table S2. Experimental design and diets used in this study.

Diet	Group	Components	*Lactobacilli* strains
Unfermented diet	Control	basal diet + 2% unfermented wheat	N/A
	Acidified Control	basal diet + 2% unfermented wheat + lactic and acetic acids	N/A
	Freeze dried Canbiocin probiotics	basal diet + 2% unfermented wheat + freeze dried Canbiocin probiotics	*L. fermentum* and *L. casei*
Fermented diet	Canbiocin probiotics	basal diet + 2% fermented wheat with Canbiocin probiotics	*L. fermentum* and *L. casei*
	Reutericyclin	basal diet + 2% fermented wheat with reutericyclin producing *Lactobacilli*	*L. reuteri* TMW1.656
	Non-reutericyclin	basal diet + 2% fermented wheat with non-reutericyclin producing *Lactobacilli* mutant	*L. reuteri* TMW1.656ΔrtcN

Figure S1. Principle coordinates analysis (PCoA) of fecal microbiota composition based on weighted UniFrac-distance of partial 16S rRNA sequences. Each dot represents individual fecal samples, colored according to the sampling age (Panel A) and according to the wheat content of feed (Panel B).

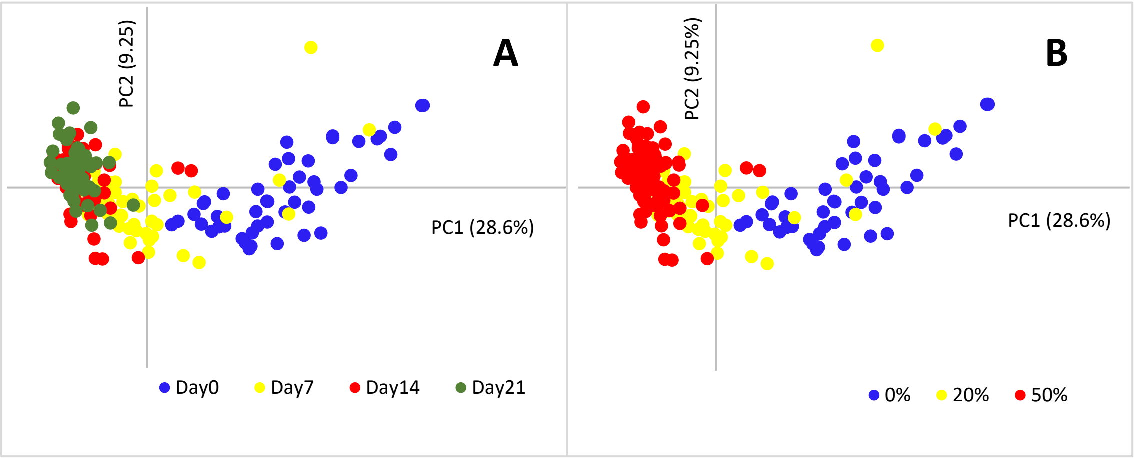

Table S3. Quality assessment of 596 bins by CheckM (* Phylogenetic affiliation were only assigned to 360 bins with completeness ≥ 70% and contamination ≤ 5%)

Bin_ID	Phylogeny *	Genome size (bp)	# contigs	N50	GC %	Completeness %	Contamination %
Bin206	unclassified *Clostridiales*	1830849	1827	28008	37.9	93.18	1.34
Bin154	*Ruminococcaceae*	1762869	1672	85982	38.5	85.68	0
Bin196	*Ruminococcus flavefaciens*	2578630	2253	34763	38.5	83.22	0
Bin155	*Ruminococcaceae*	2082788	1954	45052	39.4	95.66	0.34
Bin326	*Phascolarctobacterium*	2057086	2008	25219	38.2	98.2	0.6
Bin156	*Ruminococcaceae*	1655009	1639	15466	38.4	80.98	0
Bin33	unclassified *Bacteroidales*	2244629	1736	18188	36.6	80.59	0.74
Bin207	unclassified *Clostridiales*	1863125	1778	102122	38.4	95.97	0
Bin208	unclassified *Clostridiales*	2436536	2318	102515	36.5	94.92	1.08
Bin157	*Ruminococcaceae*	1554586	1503	66345	35.7	89.93	0.81
Bin34	unclassified *Bacteroidales*	2729792	2247	112084	39.8	95.51	0.48
Bin209	unclassified *Clostridiales*	1698269	1677	17005	36.7	93.66	0
Bin35	unclassified *Bacteroidales*	2297146	2020	24716	37.9	87.3	3.62
Bin3	*Escherichia coli*	4539067	4259	100584	36.2	98.78	0.21
Bin203	*Subdoligranulum variabile*	2419992	2241	54866	36.6	77.55	0
Bin36	unclassified *Bacteroidales*	2271448	1776	17594	38.6	80.49	0.12
Bin197	*Ruminococcus*	2105499	2009	20316	38.6	91.28	3.75
Bin348	*Desulfovibrio piger*	1636123	1660	4524	38.9	70.59	1.78
Bin210	unclassified *Clostridiales*	1732346	1649	82781	38.9	96.15	0
Bin37	unclassified *Bacteroidales*	2342019	2022	12083	36.9	86.11	1.22
Bin211	unclassified *Clostridiales*	1910205	1935	57561	37.8	90.9	0.1
Bin186	*Faecalibacterium*	2360806	2222	116677	37.7	97.54	0
Bin18	*Coriobacteriaceae*	1849683	1765	12665	36.1	91.53	1.61
Bin29	*Bacteroides coprophilus*	2770554	2322	32561	38.3	83.95	1.75
Bin32	*Alistipes putredinis*	3093702	2561	115959	36.9	99.52	0
Bin212	unclassified *Clostridiales*	2412669	2353	53548	36.4	96.37	1.88
Bin2	*Alphaproteobacteria*	1642539	1774	12239	38	95.51	0.06
Bin4	*Escherichia coli*	4045726	4109	9746	36.2	92.63	1.66
Bin213	unclassified *Clostridiales*	1623393	1767	6376	40.1	84.81	2.02
Bin355	*Pyramidobacter piscolens*	3300405	2874	65943	39.8	91.53	0
Bin31	*Bacteroides vulgatus*	4361813	3981	38325	39.1	86.72	4.48
Bin12	*Mycoplasmatales*	1431294	1527	61079	39.9	92.42	3.37
Bin149	*Lachnospiraceae*	2644724	2483	49401	40.7	95.98	0.38
Bin158	*Ruminococcaceae*	2601857	2435	301243	37.4	99.32	0.17
Bin138	*Clostridium scindens*	2885217	2986	48377	41	94.74	0.33
Bin137	*Clostridium bolteae*	4404879	4344	34725	41.7	97.22	3.39
Bin214	unclassified *Clostridiales*	1778909	1761	45079	38.2	79.87	3.69
Bin349	*Desulfovibrio piger*	2582996	2215	102585	38.8	98.7	0.59
Bin358	*Akkermansia muciniphila*	2562750	2279	23613	39	93.82	0
Bin215	unclassified *Clostridiales*	1531569	1629	12145	38.9	92.31	0
Bin359	*Akkermansia.s muciniphila*	2550919	2188	67327	36.5	92.59	1.69
Bin216	unclassified *Clostridiales*	2620615	2567	43124	38.8	95.94	4.44
Bin38	unclassified *Bacteroidales*	1927438	1643	69052	34.7	93.24	0
Bin39	unclassified *Bacteroidales*	2761614	2250	51677	37.2	95.47	3.58
Bin217	unclassified *Clostridiales*	1319117	1287	22659	39.2	87.27	0.81
Bin345	*Delftia*	3345219	3080	114904	33.6	96.84	0.67
Bin159	*Ruminococcaceae*	2341184	2273	50355	39.7	93.06	2.68
Bin193	*Ruminococcus flavefaciens*	2173514	2173	7496	38.3	90.82	1.79
Bin40	unclassified *Bacteroidales*	2701234	2204	32610	37.2	95.66	1.26
Bin218	unclassified *Clostridiales*	1996919	2051	78492	39.4	90.92	0
Bin41	unclassified *Bacteroidales*	2520126	2207	16383	38.6	88.58	3.52

Bin42	unclassified *Bacteroidales*	1842287	1694	14583	38.2	87.36	2.69
Bin357	unclassified *Opitutae*	2171368	1770	58031	34.1	93.92	0
Bin43	unclassified *Bacteroidales*	2226688	1831	12095	38.2	86.38	1.94
Bin219	unclassified *Clostridiales*	2139860	2139	85995	39.6	99.19	1.34
Bin220	unclassified *Clostridiales*	2043147	1972	53303	37.5	99.19	0
Bin13	*Mycoplasmatales*	1336820	1401	75640	39.7	95.51	0
Bin221	unclassified *Clostridiales*	1352857	1312	14366	38.5	90.28	1.4
Bin44	unclassified *Bacteroidales*	2422237	1853	37116	35.2	93.62	0
Bin222	unclassified *Clostridiales*	1119053	1213	7205	39.1	72.77	2.42
Bin45	unclassified *Bacteroidales*	2159755	1922	11314	37.3	87.2	1.07
Bin341	*Lentisphaeraceae*	2106401	2042	6125	39	78.14	3.27
Bin223	unclassified *Clostridiales*	3208704	3096	81878	38.6	96.55	4.89
Bin224	unclassified *Clostridiales*	1428668	1472	10528	39.7	85.41	4.08
Bin147	*Eubacterium rectale*	3016716	2656	46020	40.5	92.89	2.41
Bin225	unclassified *Clostridiales*	1618420	1616	33696	38.2	88.29	0.67
Bin226	unclassified *Clostridiales*	2261741	2296	60580	39.5	94.97	1.8
Bin46	unclassified *Bacteroidales*	2421738	1986	39489	34.9	96.98	0
Bin8	*Treponema*	2598084	2486	63340	39.3	99.3	0.35
Bin160	*Ruminococcaceae*	1524490	1422	44141	39.5	81.88	0
Bin47	unclassified *Bacteroidales*	2351503	1856	64818	35.8	96.71	0
Bin227	unclassified *Clostridiales*	2613503	2498	19220	37.6	92.17	3.91
Bin161	*Ruminococcaceae*	1681403	1700	36129	39.5	89.57	2.68
Bin228	unclassified *Clostridiales*	2188636	2112	109063	36.9	95.97	0.67
Bin229	unclassified *Clostridiales*	2426086	2508	60578	37.8	97.99	0.67
Bin48	unclassified *Bacteroidales*	2941455	2977	6789	37.4	78.3	2.89
Bin230	unclassified *Clostridiales*	2802820	2671	44895	37.7	97.55	0.42
Bin231	unclassified *Clostridiales*	1899796	1591	106704	36.9	98.58	0
Bin162	*Ruminococcaceae*	1766263	1634	52448	38.4	88.37	0
Bin232	unclassified *Clostridiales*	2082760	2024	75827	37.3	99.19	0
Bin49	unclassified *Bacteroidales*	2266810	1784	91382	35.5	96.54	0.75
Bin356	*Thermanaerovibrio acidaminovorans*	3327991	2971	74791	39.7	100	0
Bin233	unclassified *Clostridiales*	2396985	2302	32437	37.1	97.32	1.82
Bin50	unclassified *Bacteroidales*	3729088	3509	11025	39.1	90.78	3.04
Bin194	*Ruminococcus flavefaciens*	2237849	2286	7947	38.7	76.29	2.68
Bin51	unclassified *Bacteroidales*	3498672	3124	14590	39.2	87.95	0
Bin234	unclassified *Clostridiales*	1832603	1807	32183	40.3	99.19	2.96
Bin235	unclassified *Clostridiales*	2781264	2787	13508	37	90.43	3.9
Bin236	unclassified *Clostridiales*	1746954	1797	15807	40.4	96.1	2.15
Bin52	unclassified *Bacteroidales*	3119199	2576	38614	39.7	92.05	2.14
Bin237	unclassified *Clostridiales*	2133212	2062	73764	37.2	88.59	0.67
Bin238	unclassified *Clostridiales*	1579536	1545	83397	40.6	90.94	0.67
Bin19	*Coriobacteriaceae*	1804051	1718	13407	36.1	94.96	2.26
Bin53	unclassified *Bacteroidales*	2502981	2063	44065	36	95.48	1.3
Bin54	unclassified *Bacteroidales*	1712893	1628	9816	37.8	75.44	1.07
Bin163	*Ruminococcaceae*	2238204	2226	44483	38.6	96.98	0
Bin327	*Phascolarctobacterium*	2187908	2100	63911	37.8	99.98	0.6
Bin239	unclassified *Clostridiales*	1828019	1761	108818	36.1	86.58	0
Bin240	unclassified *Clostridiales*	2677664	2647	74195	39	81.54	0
Bin55	unclassified *Bacteroidales*	2708720	2177	29051	38.2	79.44	1.32
Bin150	*Lachnospiraceae*	3969489	3892	43630	41.3	91.14	4.75
Bin241	unclassified *Clostridiales*	1925525	1856	29444	37.2	84.34	2.01
Bin242	unclassified *Clostridiales*	2007844	1966	63047	39.9	99.19	2.15
Bin139	*Eubacteriaceae*	2181022	2255	49723	39	97.99	0.34
Bin243	unclassified *Clostridiales*	2180581	2277	64492	39.2	90.6	2.35
Bin244	unclassified *Clostridiales*	1706632	1538	111639	37.3	85.23	0
Bin245	unclassified *Clostridiales*	2489405	2376	333459	36.7	98.39	0

Bin246	unclassified *Clostridiales*	1973682	2003	15279	37	84.68	1.01
Bin151	*Lachnospiraceae*	4166508	4234	40172	39.4	94.74	3.01
Bin247	unclassified *Clostridiales*	3247545	2907	30463	38.4	96.64	4.7
Bin56	unclassified *Bacteroidales*	2740503	2362	17988	37.1	92.82	0.25
Bin248	unclassified *Clostridiales*	2205832	2206	47517	39.1	91.28	0.67
Bin249	unclassified *Clostridiales*	1931383	1908	115179	37.9	95.97	1.34
Bin57	unclassified *Bacteroidales*	2825891	2291	35700	37.2	95.47	0.13
Bin164	*Ruminococcaceae*	2700705	2571	61881	37.2	87.76	0
Bin250	unclassified *Clostridiales*	3131831	3090	89621	39.1	98.39	1.61
Bin353	*Succinatimonas*	2223481	2031	30915	39.2	87.65	2.39
Bin251	unclassified *Clostridiales*	1988121	1831	79148	38.3	92.6	1.34
Bin324	*Erysipelotrichaceae*	2205326	2115	40568	37.5	97.62	0.32
Bin252	unclassified *Clostridiales*	2481236	2266	124674	38.5	92.2	0
Bin20	*Coriobacteriaceae*	2710304	2436	200214	37.9	99.73	1.61
Bin253	unclassified *Clostridiales*	1592435	1443	79320	38.3	92.64	0.7
Bin136	*Clostridium bolteae*	3146713	3042	61365	41.3	97.47	0.63
Bin140	*Eubacterium*	2096058	1852	94653	39.8	96.45	0.95
Bin331	*Megasphaera*	2689152	2595	43150	38.2	94.31	0.6
Bin346	*Sutterella wadsworthensis*	2492678	2095	45587	33.9	97.52	1.24
Bin141	*Eubacterium*	2513023	2362	43094	39.8	94.68	0.63
Bin58	unclassified *Bacteroidales*	2086887	1704	35843	38.5	97.88	0.86
Bin330	*Phascolarctobacterium*	2170661	2130	13371	39.5	95.3	1.8
Bin254	unclassified *Clostridiales*	2482995	2189	25177	40.1	80.76	1.79
Bin255	unclassified *Clostridiales*	1970220	1878	49057	37.6	95.43	1.34
Bin256	unclassified *Clostridiales*	3029120	2799	36454	36.9	95.97	2.69
Bin191	*Ruminococcus*	2119190	2089	42295	37.7	95.99	1.41
Bin165	*Ruminococcaceae*	2451609	2347	32137	37.3	80.59	4.93
Bin142	*Eubacterium*	1699470	1770	6671	38.5	76.9	0
Bin257	unclassified *Clostridiales*	1739302	1714	6327	36.4	73.64	2.24
Bin129	*Lactobacillus johnsonii*	1907384	1930	28754	35.5	99.03	1.41
Bin258	unclassified *Clostridiales*	1888926	1812	44278	37.6	84.23	1.44
Bin347	*Dechloromonas aromatica*	1807772	1626	30276	34.8	89.57	0.47
Bin21	*Coriobacteriaceae*	1690747	1581	12055	36.2	94.83	3.13
Bin59	unclassified *Bacteroidales*	1681287	1479	11796	38	77.59	4.62
Bin259	unclassified *Clostridiales*	2936626	2740	23635	37.6	97.14	3.23
Bin260	unclassified *Clostridiales*	1293082	1395	8699	37.1	70.8	3.36
Bin204	*Subdoligranulum variabile*	2645506	2520	49156	36.5	80.25	2.04
Bin30	*Bacteroides*	5079918	4404	122658	39.2	88.84	3.41
Bin128	*Lactobacillus delbrueckii*	1477589	1451	8687	35.1	82.99	0.32
Bin340	*Veillonella*	2396989	2376	61398	38.2	99.4	2.45
Bin60	unclassified *Bacteroidales*	2910260	2518	16209	37.2	94.53	4.8
Bin261	unclassified *Clostridiales*	1266743	1367	5986	38.4	74.57	0.02
Bin354	*Succinatimonas*	1787287	1683	32177	37.5	88.79	4.18
Bin61	unclassified *Bacteroidales*	2457371	1802	18440	36.4	89.68	2.1
Bin166	*Ruminococcaceae*	2151898	2015	50019	38.1	93.88	0
Bin22	*Coriobacteriaceae*	1982127	1764	25401	36.3	98.28	3.29
Bin198	*Subdoligranulum*	2826653	2643	112415	35.6	98.3	0.87
Bin262	unclassified *Clostridiales*	1839038	1786	45360	37.7	88.95	0
Bin23	*Coriobacteriaceae*	1680601	1642	10116	39.1	90.5	2.79
Bin14	*Mycoplasmatales*	1616339	1891	30006	38.1	89.81	3.93
Bin263	unclassified *Clostridiales*	2291401	2388	7670	37.8	91.38	4.34
Bin264	unclassified *Clostridiales*	1575513	1469	33174	36.8	72.58	0
Bin265	unclassified *Clostridiales*	2531601	2366	72378	36.5	96.37	1.08
Bin266	unclassified *Clostridiales*	1879263	1986	13156	40.1	95.69	3.63
Bin267	unclassified *Clostridiales*	1622984	1614	21069	39.9	86.29	0
Bin268	unclassified *Clostridiales*	1668797	1733	8345	37.4	87.04	2.02
Bin167	*Ruminococcaceae*	2180112	2079	79115	36.1	97.99	0

Bin269	unclassified *Clostridiales*	1720739	1684	59249	39.5	90.03	0.81
Bin62	unclassified *Bacteroidales*	2977236	2328	34534	36.4	89.44	0.93
Bin9	*Treponema*	2110065	1920	22791	37.8	89.34	2.45
Bin63	unclassified *Bacteroidales*	2446683	1867	10328	37.7	75.72	2.22
Bin64	unclassified *Bacteroidales*	2267061	1753	52800	35.8	96.89	0
Bin344	*Pirellulaceae*	2332346	1705	48194	36.1	71.2	0
Bin270	unclassified *Clostridiales*	1946657	1999	33781	38.3	90.25	1.13
Bin271	unclassified *Clostridiales*	2061864	2158	65481	38.5	91.95	1.74
Bin65	unclassified *Bacteroidales*	2504743	1801	23786	37.6	73.58	0
Bin66	unclassified *Bacteroidales*	1989460	1749	26287	40.8	89.6	0.95
Bin67	unclassified *Bacteroidales*	1714397	1519	12313	40.8	81.7	4.11
Bin132	*Lactobacillus reuteri*	1496783	1473	10171	33.6	95.36	0
Bin272	unclassified *Clostridiales*	2656576	2708	66528	38.1	97.97	0
Bin342	*Lentisphaeraceae*	2078870	1625	55157	33.6	92.57	2.73
Bin187	*Faecalibacterium*	2246804	2143	35889	36.9	99.15	0.68
Bin68	unclassified *Bacteroidales*	2434754	2036	25781	36.5	96.56	2.7
Bin273	unclassified *Clostridiales*	2389425	2162	50319	38.2	74.73	3.9
Bin69	unclassified *Bacteroidales*	2322208	1840	29783	36.8	89.78	0.65
Bin274	unclassified *Clostridiales*	2261105	2150	70628	38.6	94.97	2.35
Bin130	*Lactobacillus johnsonii*	1293336	1334	42956	35.8	76.27	0
Bin10	*Treponema*	2569688	2332	81465	37.7	97.2	1.4
Bin275	unclassified *Clostridiales*	2802272	2803	66055	38	98.32	0.67
Bin70	unclassified *Bacteroidales*	2901996	2311	13684	37.3	79.43	2.75
Bin168	*Ruminococcaceae*	2047572	1877	87297	38.2	89.8	0.68
Bin328	*Phascolarctobacterium*	1465414	1561	7798	37.9	86.03	2.81
Bin143	*Eubacterium*	1881236	1888	17047	39.5	95.03	2.32
Bin276	unclassified *Clostridiales*	1969293	1902	102048	36.3	92.37	1.34
Bin71	unclassified *Bacteroidales*	2331089	1906	27233	35.9	94.76	1.09
Bin24	*Coriobacteriaceae*	2029779	1781	77820	36.4	99.19	3.51
Bin144	*Eubacterium*	1794056	1645	29179	40.8	95.77	3.55
Bin169	*Ruminococcaceae*	1697960	1738	5956	38.8	86.24	1.68
Bin205	unclassified *Clostridiales*	2131993	2073	185650	38.4	97.99	2.28
Bin277	unclassified *Clostridiales*	1897894	1922	14722	39.2	81.54	4.7
Bin192	*Ruminococcus*	2439570	2452	29950	37.3	97.99	4.87
Bin278	unclassified *Clostridiales*	2338762	2346	11343	36.8	86.35	4.81
Bin72	unclassified *Bacteroidales*	1514134	1343	15342	40.1	88.6	0.4
Bin73	unclassified *Bacteroidales*	2682180	2060	27344	38.6	90.64	4.78
Bin279	unclassified *Clostridiales*	2263384	2034	24333	39.1	95.11	3.36
Bin133	*Lactobacillus reuteri*	1886628	1840	13296	33.1	95.15	2.08
Bin5	*Escherichia coli*	4590617	4441	23061	36	97.05	1.21
Bin280	unclassified *Clostridiales*	1658702	1628	13452	40	92.34	0.34
Bin170	*Ruminococcaceae*	1700865	1782	5958	39.4	85.57	1.68
Bin332	*Megasphaera*	2278117	2230	71445	37.8	94.41	0
Bin199	*Subdoligranulum*	2928041	2799	161615	35.8	98.64	0
Bin336	*Mitsuokella*	2228453	2064	66232	34.8	86.45	0.08
Bin74	unclassified *Bacteroidales*	2484673	1793	20830	37.7	71.93	2.96
Bin75	unclassified *Bacteroidales*	2283003	1711	16966	37.1	80.96	1.23
Bin76	unclassified *Bacteroidales*	2650459	2007	25441	37.1	87.5	0.68
Bin281	unclassified *Clostridiales*	2055966	1969	25266	36.9	90.6	0.67
Bin282	unclassified *Clostridiales*	1371611	1433	9108	38.9	81.4	0
Bin283	unclassified *Clostridiales*	1198057	1313	33671	38.1	73.37	0.22
Bin15	*Mycoplasmatales*	1225896	1305	38939	40.7	94.38	3.37
Bin284	unclassified *Clostridiales*	2082480	2033	139880	36.9	90.77	0.7
Bin285	unclassified *Clostridiales*	1350016	1440	74085	38.2	74.21	0.89
Bin286	unclassified *Clostridiales*	1633266	1724	8598	40.4	94.19	2.42
Bin171	*Ruminococcaceae*	1766373	1743	11378	39.7	88.93	2.68
Bin77	unclassified *Bacteroidales*	2209579	2033	16917	38	95.86	3.39

Bin172	*Ruminococcaceae*	2032609	1797	28782	38.3	79.88	0.67
Bin287	unclassified *Clostridiales*	2719853	2651	119036	40.2	94.97	1.18
Bin78	unclassified *Bacteroidales*	2571420	2063	31496	37.9	89.56	3.46
Bin1	*Methanobrevibacter ruminantium*	2829865	3006	74937	40.4	99.2	4
Bin288	unclassified *Clostridiales*	1596527	1530	38000	40	91.16	1.57
Bin289	unclassified *Clostridiales*	2032812	1954	54383	37.6	84.45	4.7
Bin290	unclassified *Clostridiales*	2408428	2367	39986	36.4	96.37	1.08
Bin291	unclassified *Clostridiales*	2301193	2202	44239	38.8	92.06	4.99
Bin292	unclassified *Clostridiales*	1508701	1520	46168	39.6	79.48	0
Bin79	unclassified *Bacteroidales*	1728802	1594	6211	40.7	75.19	4.55
Bin80	unclassified *Bacteroidales*	2228938	1860	30674	35.6	89.31	0.88
Bin293	unclassified *Clostridiales*	2220870	2170	127927	38.3	97.99	0.84
Bin173	*Ruminococcaceae*	2753304	2574	20210	36.3	97.24	2.49
Bin174	*Ruminococcaceae*	2061437	1890	77005	39.5	97.05	0
Bin81	unclassified *Bacteroidales*	2644581	2267	11473	37.4	79.5	1.67
Bin82	unclassified *Bacteroidales*	2235881	1746	28470	35.9	94.61	1.49
Bin83	unclassified *Bacteroidales*	2173770	2099	9379	41.1	84.52	4.04
Bin145	*Eubacterium*	2021707	1983	15731	39	92.17	0.22
Bin25	*Coriobacteriaceae*	1217896	1272	5679	38.3	74.84	2.42
Bin84	unclassified *Bacteroidales*	2661534	2182	34087	36.5	91.12	3.21
Bin85	unclassified *Bacteroidales*	2290494	1976	11344	37.2	80.6	1.07
Bin86	unclassified *Bacteroidales*	2515548	2157	55863	35	86.54	0.69
Bin175	*Ruminococcaceae*	1372327	1374	42269	38.6	73.94	0.34
Bin87	unclassified *Bacteroidales*	2639495	2368	15850	38.6	88.29	0.19
Bin176	*Ruminococcaceae*	2518734	2617	24385	39.7	91.87	1.48
Bin88	unclassified *Bacteroidales*	1540644	1411	24417	40.4	75.37	0.48
Bin89	unclassified *Bacteroidales*	1656522	1426	15993	39.1	71.6	0
Bin90	unclassified *Bacteroidales*	2130712	1764	17352	36.3	74.18	1.67
Bin200	*Subdoligranulum*	2001516	1874	15682	35.6	92.78	2.61
Bin294	unclassified *Clostridiales*	1755699	1699	19784	39.9	90.53	1.36
Bin91	unclassified *Bacteroidales*	2996947	3051	27733	38.7	81.57	3.67
Bin92	unclassified *Bacteroidales*	2210670	2088	6682	37.3	83.34	1.13
Bin93	unclassified *Bacteroidales*	3309932	2960	19987	37.6	94.85	2.1
Bin94	unclassified *Bacteroidales*	1998319	1785	5581	36.4	73.15	2.94
Bin95	unclassified *Bacteroidales*	2896936	2689	34827	39.1	94.49	4.15
Bin295	unclassified *Clostridiales*	1747512	1751	93699	40.4	90.94	0.84
Bin96	unclassified *Bacteroidales*	1972060	1660	54107	41.5	85.56	1.54
Bin337	*Mitsuokella*	2227532	2060	20606	39.2	96.54	2.53
Bin360	*Unclassidied TM7*	2269311	1996	14377	37.4	74.36	3.08
Bin97	unclassified *Bacteroidales*	1824678	1592	12148	38.2	91.78	3.92
Bin296	unclassified *Clostridiales*	1734705	1714	41258	37.1	90.27	0.5
Bin125	*Lactobacillus amylovorus*	2092575	2282	17389	35.4	96.56	2.19
Bin134	*Lactobacillus reuteri*	1837333	1898	21850	34.4	95.99	0.55
Bin98	unclassified *Bacteroidales*	1854803	1591	17914	40.6	80.19	0.24
Bin195	*Ruminococcus flavefaciens*	2924649	2704	29607	38.7	90.94	0.06
Bin351	*Myxococcales*	3174537	2385	20315	36.7	76.65	1.77
Bin297	unclassified *Clostridiales*	1894814	1864	56400	38.8	90.81	0.34
Bin298	unclassified *Clostridiales*	1754008	1780	22966	36.1	82.66	0.22
Bin131	*Lactobacillus johnsonii*	2354155	2481	22259	36.1	97.77	3.54
Bin177	*Ruminococcaceae*	2189092	2103	47065	39.3	97.27	0
Bin99	unclassified *Bacteroidales*	2248797	1866	34014	40.6	93.65	0.21
Bin329	*Phascolarctobacterium*	1335061	1402	6254	37.9	74.09	2.84
Bin100	unclassified *Bacteroidales*	1996743	1652	7938	35.4	76.95	3.04
Bin178	*Ruminococcaceae*	2132237	2012	59826	36.3	97.99	0
Bin101	unclassified *Bacteroidales*	2086320	1717	19705	36.1	70.02	1.85
Bin152	*Lachnospiraceae*	2294616	2307	16503	39	87.87	4.35

Bin352	*Succinivibrionaceae*	2691173	2290	21390	39.9	94.49	2.11
Bin299	unclassified *Clostridiales*	2379421	2445	11275	36.9	90.25	3.02
Bin102	unclassified *Bacteroidales*	2131512	1807	16617	35.7	84.68	4.41
Bin148	*Lachnospiraceae*	2241106	1906	73454	36.5	96.38	0
Bin103	unclassified *Bacteroidales*	1875526	1663	5248	36.7	77.5	2.67
Bin26	*Coriobacteriaceae*	2012174	1990	8524	36.8	87.12	3.84
Bin300	unclassified *Clostridiales*	1446482	1501	8285	37	70.38	1.34
Bin135	*Clostridiaceae*	2321535	2307	15848	39.7	91.85	4.43
Bin27	*Coriobacteriaceae*	1859870	1738	20475	37.5	94.76	0.44
Bin301	unclassified *Clostridiales*	2620908	2503	8128	35.9	86.75	3.85
Bin302	unclassified *Clostridiales*	1762539	1742	46932	40.1	90.92	2.68
Bin104	unclassified *Bacteroidales*	2043352	1734	17974	39.5	81.95	1.35
Bin105	unclassified *Bacteroidales*	2115922	1845	18073	34.8	86.51	1.28
Bin106	unclassified *Bacteroidales*	3360894	2818	45863	39.6	91.95	1.3
Bin179	*Ruminococcaceae*	1733732	1623	35547	38.6	87.7	0
Bin303	unclassified *Clostridiales*	1522075	1530	9711	36.9	71.1	3.91
Bin304	unclassified *Clostridiales*	2277679	2081	68008	37.8	96.61	0
Bin6	*Escherichia coli*	4378339	4166	56008	36.2	98.1	0.51
Bin305	unclassified *Clostridiales*	1556708	1484	22206	39.6	87.88	0.81
Bin107	unclassified *Bacteroidales*	2422660	1843	17841	37.2	86.9	2.04
Bin338	*Mitsuokella*	2584130	2609	29763	38	83.51	1.83
Bin108	unclassified *Bacteroidales*	2212806	1742	8802	36.5	84.39	2.92
Bin306	unclassified *Clostridiales*	1931836	1760	14049	36.8	91.89	1.8
Bin153	*Lachnospiraceae*	2396817	2496	19514	38.9	91.24	2.86
Bin307	unclassified *Clostridiales*	2977250	2889	38767	38.5	97.55	4.36
Bin109	unclassified *Bacteroidales*	2039448	1844	20369	40.8	82.77	1.67
Bin126	*Lactobacillus amylovorus*	1872704	2024	21308	35.9	93.1	3.63
Bin11	*Treponema*	3713748	3443	23790	37.8	92.83	4.46
Bin180	*Ruminococcaceae*	1184430	1215	14014	38.3	72.62	3.8
Bin181	*Ruminococcaceae*	1370630	1410	6479	39.7	73.17	1.51
Bin127	*Lactobacillus amylovorus*	2305390	2673	7851	35.5	71.87	4.85
Bin308	unclassified *Clostridiales*	2776932	2584	9135	37.7	80.16	3.76
Bin309	unclassified *Clostridiales*	1485439	1472	53265	39.1	71.14	0.67
Bin110	unclassified *Bacteroidales*	2318070	1921	34010	34.4	93.21	0.75
Bin339	*Mitsuokella multacida*	2280648	2057	73000	36.8	99.07	0.23
Bin16	*Mycoplasmatales*	1360808	1457	85127	39.7	89.16	2.81
Bin111	unclassified *Bacteroidales*	1831141	1544	14930	36.7	90.49	0.81
Bin112	unclassified *Bacteroidales*	2036153	1766	18811	37.9	74.03	1.79
Bin310	unclassified *Clostridiales*	1569787	1638	15266	37.2	88.96	4.88
Bin311	unclassified *Clostridiales*	1403937	1349	9202	38.4	72.66	2.01
Bin188	*Faecalibacterium*	1692888	1597	25270	37	86.52	2.63
Bin350	*Desulfovibrio piger*	2137733	1868	21919	38.7	88.94	0.59
Bin7	*Escherichia coli*	3690123	3667	9802	36.1	81.73	0.75
Bin312	unclassified *Clostridiales*	1431094	1344	19208	36.5	70.94	4.36
Bin113	unclassified *Bacteroidales*	1797376	1739	5314	41.2	73.41	4.48
Bin333	*Megasphaera*	2626568	2595	52653	37.9	96.31	0.81
Bin313	unclassified *Clostridiales*	1985734	2068	45508	38.6	89.85	2.85
Bin189	*Faecalibacterium*	2148852	2039	32749	36.9	97.49	2.35
Bin314	unclassified *Clostridiales*	2648966	2871	19098	37.3	96.31	0.67
Bin201	*Subdoligranulum*	1964712	1779	58535	35.7	95.15	0
Bin114	unclassified *Bacteroidales*	1783367	1600	10225	40.6	77.19	2.14
Bin182	*Ruminococcaceae*	1826106	1887	13823	39.4	90.02	2.68
Bin315	unclassified *Clostridiales*	1658327	1638	17084	37.3	80.33	4.92
Bin146	*Eubacterium*	2527670	2706	6884	38.7	83.24	3.24
Bin115	unclassified *Bacteroidales*	2210555	1714	22129	37.1	75.05	4.26
Bin316	unclassified *Clostridiales*	1446827	1562	7106	40.1	88.72	0.81
Bin317	unclassified *Clostridiales*	1811894	1782	6272	36.9	87.38	2.24

Bin116	unclassified *Bacteroidales*	2337932	2206	19912	36.3	92.21	4.3
Bin117	unclassified *Bacteroidales*	2190512	2047	18531	41.5	82.96	3.31
Bin118	unclassified *Bacteroidales*	1923735	1995	7304	40.7	79	4.31
Bin183	*Ruminococcaceae*	1406707	1453	21106	38.5	74.61	2.01
Bin334	*Megasphaera*	2241385	2084	49478	38	98.3	0.06
Bin184	*Ruminococcaceae*	2048608	1914	79805	36.6	91.95	0.67
Bin343	*Lentisphaeraceae*	2004189	1701	23419	37.5	86.55	1.35
Bin119	unclassified *Bacteroidales*	1964288	1745	14945	41.7	86.29	2.57
Bin318	unclassified *Clostridiales*	2153926	2062	61047	38.6	96.98	1.12
Bin319	unclassified *Clostridiales*	1343990	1432	6359	38.3	84.02	2.8
Bin320	unclassified *Clostridiales*	1796414	1906	14183	36.1	89.71	0.34
Bin120	unclassified *Bacteroidales*	2333386	1866	24877	37	87.22	3.43
Bin121	unclassified *Bacteroidales*	2067332	1911	31884	38.1	94.34	1.08
Bin190	*Faecalibacterium*	1941345	1912	26562	36.9	95.58	4.55
Bin185	*Ruminococcaceae*	2281111	2023	17389	36.2	96.71	0
Bin335	*Megasphaera*	2010916	1896	31707	38	96.77	0.12
Bin321	unclassified *Clostridiales*	2124251	2120	12928	36.9	87.76	4.42
Bin322	unclassified *Clostridiales*	1645263	1730	7880	39.2	76.17	1.98
Bin28	*Coriobacteriaceae*	1696648	1711	6161	36.5	72.53	4.49
Bin325	*Erysipelotrichaceae*	2212377	2170	35697	38	89.52	3.17
Bin17	*Mycoplasmatales*	1819014	2104	47690	40	91.29	3.68
Bin323	unclassified *Clostridiales*	1311736	1539	6551	37.4	73.97	0.67
Bin122	unclassified *Bacteroidales*	2259638	1814	20780	36.1	95.46	1.35
Bin202	*Subdoligranulum*	1999819	1919	10853	35.4	86.37	2.04
Bin123	unclassified *Bacteroidales*	2100023	1942	8678	40.5	77.07	3.61
Bin124	unclassified *Bacteroidales*	1873840	1589	6907	36.3	70.96	4.28
Bin361		1310798	1491	6067	36.3	69.22	2.46
Bin362		2973976	3198	11671	37.9	69.08	9.92
Bin363		2468156	1917	14979	37.6	68.92	0.37
Bin364		2233528	2166	8986	38.9	68.9	5.64
Bin365		1603743	1515	135488	37.3	68.6	0
Bin366		1129220	1317	8837	39.5	68.46	2.25
Bin367		2562344	2478	29474	38.2	68.28	2.15
Bin368		1854379	1923	4949	39.1	68.23	5.14
Bin369		2167306	1648	20958	40.5	68.1	0
Bin370		1694210	1815	13649	40.3	67.94	8.95
Bin371		1503394	1597	4467	38.5	67.93	9.55
Bin372		2002013	1867	5452	35.2	67.82	4.14
Bin373		2021820	1856	6342	39.3	67.72	6.54
Bin374		1791261	1951	5299	38.8	67.65	5.48
Bin375		1365044	1532	4677	40.2	67.52	7.27
Bin376		1934432	1818	5647	36.9	67.5	2.83
Bin377		1153419	1198	4548	39.4	67.48	1.96
Bin378		1334982	1358	6963	38.5	67.4	3.02
Bin379		2408349	2434	6818	38.9	67.24	6.9
Bin380		1254731	1319	4818	39.7	67.21	2.24
Bin381		1102516	1163	5485	38.5	67.01	3.37
Bin382		1212776	1346	3911	39.7	66.78	5.43
Bin383		1856568	1620	10874	37.2	66.51	2.71
Bin384		1642157	1714	3888	40.7	66.45	6.38
Bin385		1535590	1609	6355	37.8	66.38	1.72
Bin386		2398121	1952	14140	36.6	66	3.83
Bin387		1506353	1587	11286	39.6	65.88	0
Bin388		2440555	2614	3811	39	65.86	6.88
Bin389		1659016	1442	36082	37	65.66	2.01
Bin390		1511610	1660	5072	36.8	65.07	5.12
Bin391		1965207	1772	39194	39.5	65	0.31

Bin392	1232678	1261	20585	39.3	64.89	1.61
Bin393	2139668	1804	10600	37.3	64.66	0
Bin394	2172764	1664	27278	35.3	64.66	0
Bin395	1056935	1081	4378	39	64.48	0
Bin396	1044913	1046	5361	39.4	64.43	4.15
Bin397	1120639	1149	5002	39	63.81	0.87
Bin398	1524997	1461	74311	40.1	63.76	1.51
Bin399	1315030	1456	4306	38	63.6	5.27
Bin400	1252940	1425	4289	40	63.53	8.9
Bin401	2030149	1934	28568	39.2	63.5	7.52
Bin402	2285915	2649	4969	38.2	63.44	7.27
Bin403	1063973	1091	836454	34.9	63.25	0.85
Bin404	1302128	1449	7144	34.7	63.22	8.83
Bin405	1193006	1216	5177	38.7	63.21	0.16
Bin406	1357436	1402	6822	36.7	63.11	5.09
Bin407	1107204	1206	3296	38.2	63.07	5.78
Bin408	1336841	1361	7767	39.5	63.05	2.42
Bin409	1306560	1345	8121	39.6	62.74	3.02
Bin410	1410508	1508	5069	38	62.6	0
Bin411	2039121	1502	37377	36	62.41	0.37
Bin412	1428703	1406	5019	36.5	62.36	2.95
Bin413	1857618	2022	8047	39.5	62.07	6.9
Bin414	3195805	3548	17061	37.2	62.03	8.98
Bin415	2077190	1553	9600	37.8	61.92	0.19
Bin416	1749183	1859	13581	40.1	61.89	3.02
Bin417	1459340	1610	6369	37.3	61.51	0.67
Bin418	1703018	1854	25477	37.3	61.3	8.35
Bin419	1267823	1405	3491	39.3	60.84	4.5
Bin420	2144216	2460	3585	38.5	60.8	5.24
Bin421	1519910	1514	69773	36.8	60.74	9.4
Bin422	1525945	1310	7972	37	60.65	2
Bin423	1967855	1657	4061	37.7	60.46	8.87
Bin424	1223863	1515	5767	40	60.29	7.72
Bin425	1656526	1734	7527	36.7	60.06	3.45
Bin426	2277586	2402	8574	40.5	60.05	3.55
Bin427	981984	1066	4128	38.4	60.05	0.37
Bin428	1288927	1406	4986	37.6	59.99	0.81
Bin429	1843394	1622	13816	36.8	59.84	0.38
Bin430	1130162	1237	7522	38.9	59.74	1.68
Bin431	1909114	1968	3645	40.7	59.71	8.65
Bin432	1272744	1229	7493	40.5	59.67	1.9
Bin433	1808196	1674	4909	39.2	59.38	6.09
Bin434	1123184	1154	5452	38.4	59.32	3.19
Bin435	1830656	1883	12955	36.8	58.93	6.25
Bin436	2043955	1660	16959	36.5	58.71	4.83
Bin437	2064895	1928	16798	39.1	58.62	0
Bin438	1304382	1359	8412	37	58.62	0
Bin439	1122848	1199	4205	40.3	58.57	0.4
Bin440	1447039	1424	9281	34.8	58.18	1.13
Bin441	2223136	1955	17178	38.2	58.02	4.25
Bin442	1293223	1438	3945	37.8	57.96	5.11
Bin443	1090948	1229	3835	40.4	57.89	5.26
Bin444	1200336	1262	5428	35.8	57.78	1.34
Bin445	2722530	2478	7284	38.5	57.76	1.72
Bin446	893826	903	14221	38	57.76	6.11
Bin447	1909199	1522	6579	36.9	57.68	8.54
Bin448	1316469	1514	4241	40.8	57.47	5.17

Bin449	2035486	1811	12610	37.7	57.36	7.59
Bin450	2092590	1928	8112	37.5	57.24	5.17
Bin451	1682184	1750	6544	40.4	57.06	4.71
Bin452	2365437	2292	5550	38.9	56.99	2
Bin453	1157956	1262	3910	37.5	56.61	0.81
Bin454	1095620	1119	3432	35.9	56.54	2.96
Bin455	1627873	1919	5148	38.8	56.53	1.59
Bin456	1072942	1190	4897	39.7	56.33	1.43
Bin457	744099	764	92760	34.8	55.74	3.7
Bin458	999476	1026	16143	40	55.65	0.7
Bin459	824290	917	27926	34.8	55.56	1.28
Bin460	961899	1030	11126	38.4	55.52	0
Bin461	1744531	1932	5603	36.5	55.45	8
Bin462	1577161	1363	4639	38.9	55.29	3.62
Bin463	1471716	1336	32785	39.6	54.83	2.01
Bin464	1044685	1110	5323	38.9	54.66	1.72
Bin465	1430451	1550	3388	39.9	54.58	9.11
Bin466	1664482	1854	4840	38.9	54.39	3.51
Bin467	2396819	2601	6838	36.9	54.37	9.28
Bin468	956092	997	3877	38.7	54.21	0.48
Bin469	1151845	1018	18326	36	54.16	0
Bin470	1353123	1450	6502	40.2	54.02	5.17
Bin471	1365045	1425	4217	36.6	54	3.77
Bin472	842914	883	6381	40.2	53.99	2.24
Bin473	1592989	1657	3434	40.6	53.87	5.34
Bin474	1464852	1649	6949	39.8	53.79	6.9
Bin475	1166255	1352	4345	39	53.68	7.48
Bin476	1110080	1169	3864	38.5	53.64	4.64
Bin477	1015727	1000	11865	37.7	53.61	9.48
Bin478	1364517	1394	40544	37	53.5	1.68
Bin479	2036698	1468	16371	37.6	53.45	0
Bin480	1075416	1293	4199	38.1	53.19	2.06
Bin481	1145424	1310	3767	37.9	52.9	5.17
Bin482	1408638	1398	5317	36.9	52.71	5.26
Bin483	953388	955	70335	40.6	52.66	0
Bin484	1606183	1547	3686	35.4	52.58	4.26
Bin485	1845347	1502	6534	37.7	52.35	0
Bin486	1839645	1864	4762	37.7	51.72	3.45
Bin487	999395	1130	4498	39.3	51.72	1.72
Bin488	1854460	1688	7261	39.2	51.65	0.86
Bin489	1476623	1456	3224	36.6	51.23	6.02
Bin490	2003427	2397	3148	38.7	51.22	7.86
Bin491	1686034	1759	4743	39	51.03	1.51
Bin492	1108364	1316	3442	39.1	51.01	3.6
Bin493	1017259	1054	12793	38.4	51.01	3.69
Bin494	1139929	1254	13351	37.3	50.99	4.7
Bin495	2194692	2423	3257	38.9	50.92	8.39
Bin496	1173324	1218	5158	38.2	50.17	1.72
Bin497	1866523	2090	3858	35.8	50.16	7.14
Bin498	1126168	1359	3610	38.5	50.03	0.84
Bin499	3277796	2985	32767	38	99.14	5.02
Bin500	1966701	1981	9973	38.3	85.7	5.02
Bin501	2240957	2115	12341	39.8	76.72	5.02
Bin502	1793018	1764	27158	39.2	77.56	5.15
Bin503	1976925	1801	7832	36.9	75.22	5.16
Bin504	1878262	1807	10672	36.3	98.28	5.17
Bin505	1426083	1421	8193	33.2	70.69	5.17

Bin506	2662936	2432	85913	38.8	92.34	5.2
Bin507	2139673	2220	9920	38.8	79.86	5.21
Bin508	1637023	1743	4711	37.3	79.79	5.24
Bin509	2372517	1857	16259	36.4	76.5	5.25
Bin510	2598569	2210	54716	36.6	94.83	5.33
Bin511	1824212	1881	12493	38.9	87.19	5.37
Bin512	2136769	1883	52688	35.6	88.64	5.49
Bin513	2459858	2223	61970	37.1	97.58	5.5
Bin514	1918557	1827	4882	37.1	70.86	5.53
Bin515	1983956	1854	11355	38.1	92.92	5.54
Bin516	2411490	2409	14271	37	91.44	5.59
Bin517	1152004	1307	9552	38.6	75.87	5.62
Bin518	2291540	1748	63204	37.2	71.22	5.66
Bin519	2573945	2477	99043	36.9	100	5.68
Bin520	2466033	2084	10316	36.6	85.85	5.72
Bin521	2241228	2136	25545	37.7	94.46	5.74
Bin522	1741172	1637	19547	40.3	87.7	5.76
Bin523	3589326	3446	54690	38.5	96.86	5.77
Bin524	1740354	1747	15838	36.8	75.62	5.81
Bin525	2571745	2634	10356	37.8	89.28	5.83
Bin526	2200911	2154	14936	37	94.95	5.88
Bin527	1966188	1917	7969	38.1	81.17	5.91
Bin528	1799007	1749	16035	36.8	73.23	5.91
Bin529	2332343	2481	14957	37.4	87.27	5.98
Bin530	1378029	1432	7085	39.5	78.13	5.99
Bin531	1600341	1603	4978	39	71.3	6.03
Bin532	4164407	4078	81930	38	97.99	6.04
Bin533	1343577	1506	6698	37	74.13	6.04
Bin534	1238049	1302	57401	38.4	92.13	6.18
Bin535	1717864	1820	9895	40.1	98.19	6.26
Bin536	1960524	1855	16968	35.4	92.03	6.46
Bin537	1751733	1780	13736	36.9	91.72	6.54
Bin538	4104711	4144	61491	38.4	98.55	6.57
Bin539	1878582	1836	30545	39.7	79.98	6.6
Bin540	2185120	2110	42883	38.6	95.34	6.61
Bin541	1929332	1942	6011	36.9	84.82	6.62
Bin542	1520479	1576	41939	40.2	84.83	6.63
Bin543	3287706	3151	16655	38.7	94.13	6.66
Bin544	3054476	3236	13676	35.6	90.73	6.72
Bin545	2717093	2490	9993	35.8	93.01	6.76
Bin546	1776760	1937	11635	38.9	80.98	6.83
Bin547	1942522	2176	9039	39	78.61	6.94
Bin548	1706268	1800	10885	38.2	79.25	6.96
Bin549	1764100	1855	4871	37	76.71	7.03
Bin550	1954045	1846	62478	39.5	90.23	7.05
Bin551	1545271	1691	5163	40	77.74	7.05
Bin552	1937801	1941	16548	36.6	91.61	7.13
Bin553	1955867	1803	7391	40.7	74.36	7.14
Bin554	2106644	1950	28768	36.9	94.74	7.18
Bin555	3258633	2713	63761	37.5	99.02	7.24
Bin556	2299186	2301	9415	40.4	83.44	7.27
Bin557	4074606	4164	27117	41.1	87.97	7.34
Bin558	1570448	1724	7931	40.3	98.28	7.37
Bin559	2155938	2049	7414	41	85.64	7.57
Bin560	1759147	1611	11959	40.6	84.43	7.59
Bin561	2177353	2360	5738	39.6	75.56	7.65
Bin562	1839667	1827	8586	38.1	87.15	7.66

Bin563	2490848	2247	22329	36.8	89.66	7.68
Bin564	3321712	2936	9454	37.4	89.72	7.69
Bin565	1362611	1281	16653	40.7	95.51	7.72
Bin566	2530947	2023	44585	34.3	93.1	7.84
Bin567	2550816	2698	19736	38.8	86.77	7.88
Bin568	1809625	1993	10322	39.3	82.62	7.9
Bin569	1702724	2116	4591	38.8	76.16	8.04
Bin570	2376943	2332	60127	39.3	97.32	8.22
Bin571	3694456	3203	49266	37.2	95.66	8.33
Bin572	2130877	2048	4929	40.1	73.07	8.35
Bin573	2198409	2063	45884	37	92.66	8.45
Bin574	2185721	1714	20828	35.5	89.76	8.49
Bin575	2341095	2108	10185	40.5	88.79	8.57
Bin576	3237146	3196	28485	39.1	80.76	8.63
Bin577	2816250	2525	11911	36.8	92.83	8.65
Bin578	1715919	1822	7148	36.8	71.46	8.67
Bin579	3354197	3573	19203	40.3	93.57	8.89
Bin580	1952089	1881	4954	40.5	73.81	8.93
Bin581	2952581	2721	21077	38.5	94.75	8.96
Bin582	2439996	2562	15432	37.7	91.72	9.06
Bin583	2534311	2460	59550	38.8	94.97	9.08
Bin584	2544605	2377	17895	39.7	88.62	9.16
Bin585	2739071	2851	30885	39.6	99.11	9.37
Bin586	2386437	2244	8616	40.6	86.97	9.38
Bin587	2051823	1759	7224	35.2	73.09	9.38
Bin588	3682531	4100	15862	37.9	89.07	9.4
Bin589	1826271	1831	17189	37.9	79.31	9.4
Bin590	2153349	2084	52811	37.9	83.05	9.53
Bin591	2624229	2313	21667	36	94.88	9.59
Bin592	2189278	1994	12534	36.8	92.66	9.63
Bin593	2040605	1931	8579	35.9	92.21	9.68
Bin594	2077685	2073	32982	37.9	92.89	9.73
Bin595	4004257	3782	43413	38.6	96.19	9.92
Bin596	2310127	2396	13485	37.5	91.11	9.98

Table S4. Accession number and reference to the biochemical characterization of enzymes blast for starch, fructan and lactose degradation

Name	Protein	UniProt accession #	Reference
Amy1_Bat	Beta/alpha-amylase	A0A2N0UIC8	[1]
Amy4_Bat	Alpha-amylase	A0A2N0UX89	[1]
Amy12_Fp	Pullulanase	A0A2N0UU23	[1]
GlgB_Fic	1,4-alpha-glucan branching enzyme GlgB	P30539	[2]
susG_Bt	Neopullulanase SusG	Q8A1G3	[3]
susA_Bat	Neopullulanase SusA	Q8A1G0	[3]
susB_Bat	Glucan 1,4-alpha-glucosidase SusB	G8JZS4	[3]
BT_3082_Bat	2,6-beta-D-fructofuranosidase	Q8A373	[4]
BT_1765_Bat	Levanase (2,6-beta-D-fructofuranosidase)	Q8A6W1	[4]
BT_1760_Bat	Glycoside hydrolase family 32	Q8A6W6	[4]
BT_1754_Bat	Two-component system sensor histidine kinase/response regulator	Q8A6X1	[4]
inuJ_Lb	Inulosucrase	Q74K42	[5]
sacA_Fic	Sucrose-6-phosphate hydrolase	A0A173R035	[6]
scrB_Bs	β-fructofuranosidase	D0R647	[7]
LacA_Lb	Beta-galactosidase LacA	C6H178	[8]
BbgI_Fic	Beta-galactosidase BbgII	D4QFE6	[9]
BbgIII_Fic	Beta-galactosidase BbgIII	A4K5H9	[9]
BbgIV_Fic	Beta-galactosidase BbgIV	D4QFE8	[9]
LacM_Lb	Beta-galactosidase LacM	Q02604	[8]

FigureS1

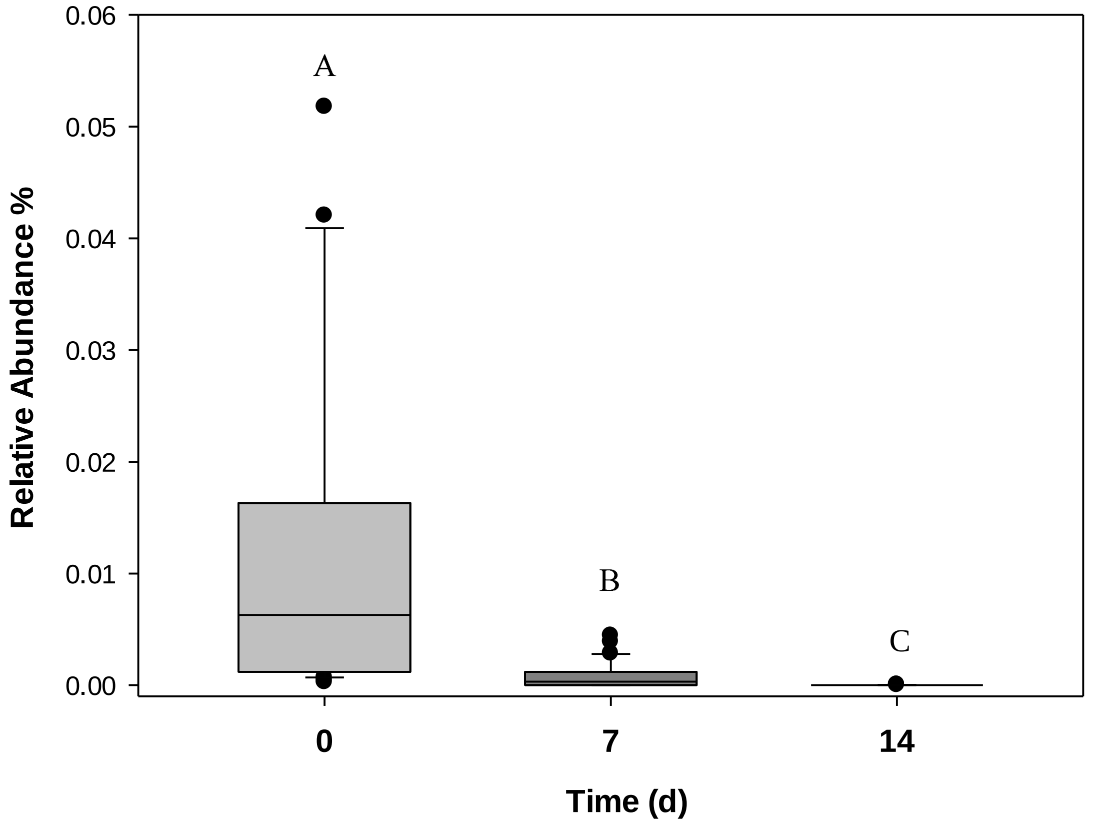

Reference

1. Ze X, David YB, Laverde-Gomez JA, Dassa B, Sheridan PO, et al. Unique organization of extracellular amylases into amylosomes in the resistant starch-utilizing human colonic Firmicutes bacterium *Ruminococcus bromii.* MBio.2015;6:e01058-15.
2. Rumbak E, Rawlings D, Lindsey G and Woods D. Characterization of the *Butyrivibrio fibrisolvens* glgB gene, which encodes a glycogen-branching enzyme with starch-clearing activity. J Bacteriol.1991;173:6732-41.
3. Martens EC, Koropatkin NM, Smith TJ and Gordon JI. Complex glycan catabolism by the human gut microbiota: the Bacteroidetes Sus-like paradigm. J Biol Chem.2009;284:24673-7.
4. Sonnenburg ED, Zheng H, Joglekar P, Higginbottom SK, Firbank SJ, et al. Specificity of polysaccharide use in intestinal *Bacteroides* species determines diet-induced microbiota alterations. Cell.2010;141:1241-52.
5. Pijning T, Anwar MA, Böger M, Dobruchowska JM, Leemhuis H, et al. Crystal structure of inulosucrase from *Lactobacillus*: insights into the substrate specificity and product specificity of GH68 fructansucrases. J Mol Biol.2011;412:80-93.
6. Chen C, Zhou F, Ren J, Ai L, Dong Y, et al. Cloning, expression and functional validation of a β-fructofuranosidase from *Lactobacillus plantarum.* Process Biochem.2014;49:758-67.

7. Yin X, Heeney DD, Srisengfa YT, Chen S-Y, Slupsky CM, et al. Sucrose metabolism alters *Lactobacillus plantarum* survival and interactions with the microbiota in the digestive tract. FEMS Microbiol Ecol.2018;94.
8. Schwab C, Sørensen KI and Gänzle MG. Heterologous expression of glycoside hydrolase family 2 and 42 β-galactosidases of lactic acid bacteria in *Lactococcus lactis.* Syst Appl Microbiol.2010;33:300-7.
9. Goulas TK, Goulas AK, Tzortzis G and Gibson GR. Molecular cloning and comparative analysis of four β-galactosidase genes from *Bifidobacterium bifidum* NCIMB41171. Appl Microbiol Biotechnol.2007;76:1365-72.
